# Correction: Structural Characterization of the *Saccharomyces cerevisiae* THO Complex by Small-Angle X-Ray Scattering

**DOI:** 10.1371/journal.pone.0106604

**Published:** 2014-08-25

**Authors:** The PLOS ONE Staff

## Notice of Republication

This article was republished on July 1st, 2014, due to formatting errors in the Materials and Methods section of the text. The publisher apologizes for this error. Please download the PDF again to view the corrected article. The originally published, uncorrected article and the republished, corrected article are provided here for reference.

## Supporting Information

File S1 Originally published, uncorrected article.
(PDF)

File S2 Republished, corrected article.
(PDF)
