# Supplementary figures and images for: Structural Characterization of the Saccharomyces cerevisiae THO Complex by Small-Angle X-Ray Scattering
Source: PLoS One. 2014 Jul 25;9(7):e103470. doi: 10.1371/journal.pone.0103470 (PMC4111604; doi:10.1371/journal.pone.0103470)

Figure S1

A

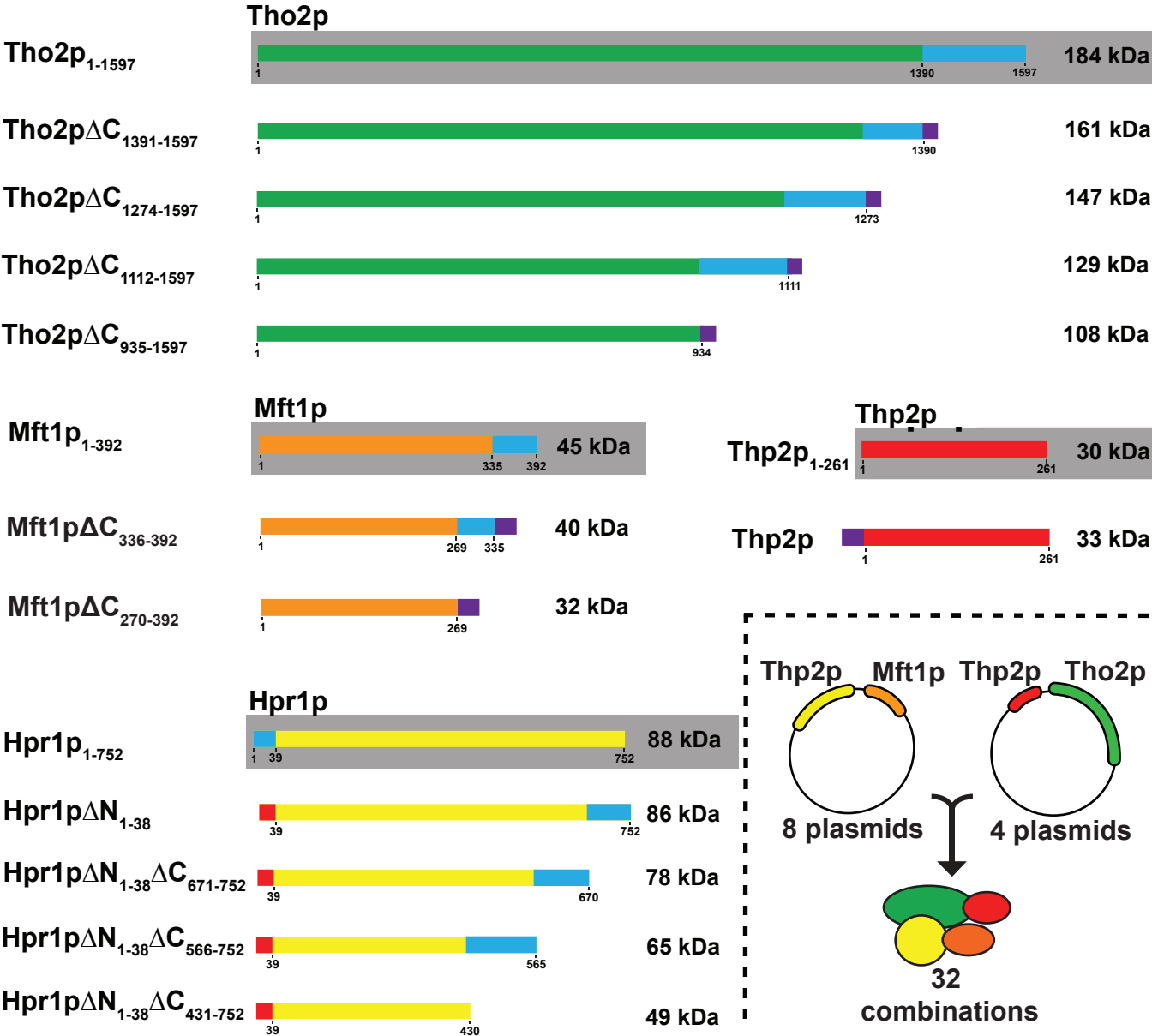

B

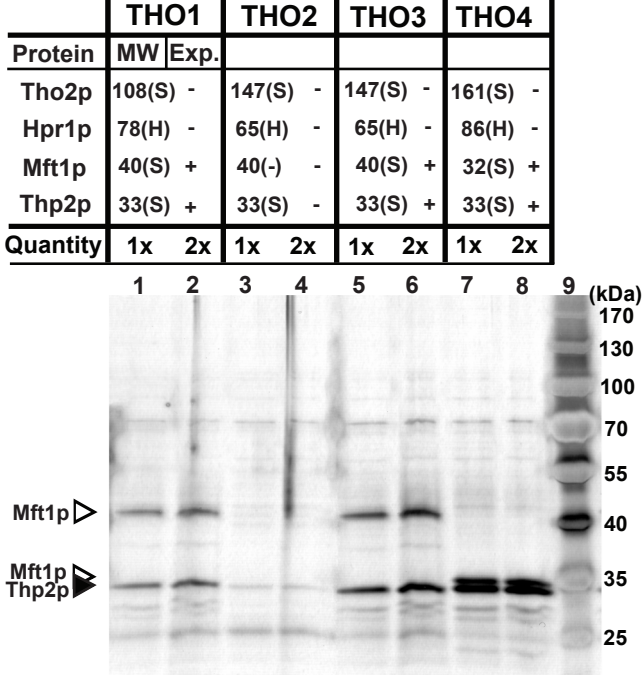

C

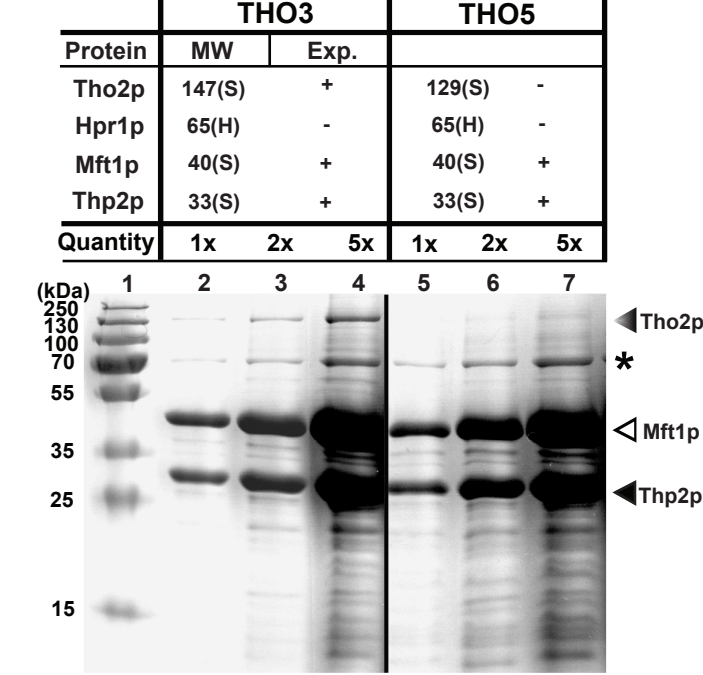

Supplement: Figure S1 — Overview of constructs, truncations and combinations of the THO complex. A. Overview of Tho2p, Hpr1p, Mft1p, and Thp2p truncation variants. The proteins were named according to their full-length form (grey boxes). Blue boxes: stretch of residues removed in the construct directly below. Purple boxes: Strep II Tag. Red boxes: 6xHis-Tag. Expected molecular weights based on sequence are indicated. Inset: Combination of two plasmids during co-transformation yields a total of 32 combinations of the THO complex. Proteins and genes were coloured as in A. B. 10 µg (1x) or 20 µg (2x) of final eluates of THO1-4 as analysed by Coomassie-stained SDS-PAGE (lanes 1–8). Lane 9: Marker proteins of indicated molecular weights. C. 20–100 µg of final eluates of THO3 and THO5 analysed by Coomassie-stained SDS-PAGE (lanes 2–7). Lane 1: Marker proteins of indicated molecular weights. In both B and C, the expected protein molecular weight (MW), the fusion tag (H: 6xHis-Tag, S: Strep II Tag) and whether that protein is expressed (+/-) are indicated. Arrowheads: black fill, Thp2p; white fill, Mft1p; gradient, Tho2p; asterisk, a protein contaminant. (PDF) [file pone.0103470.s001.pdf]

Figure S2

A

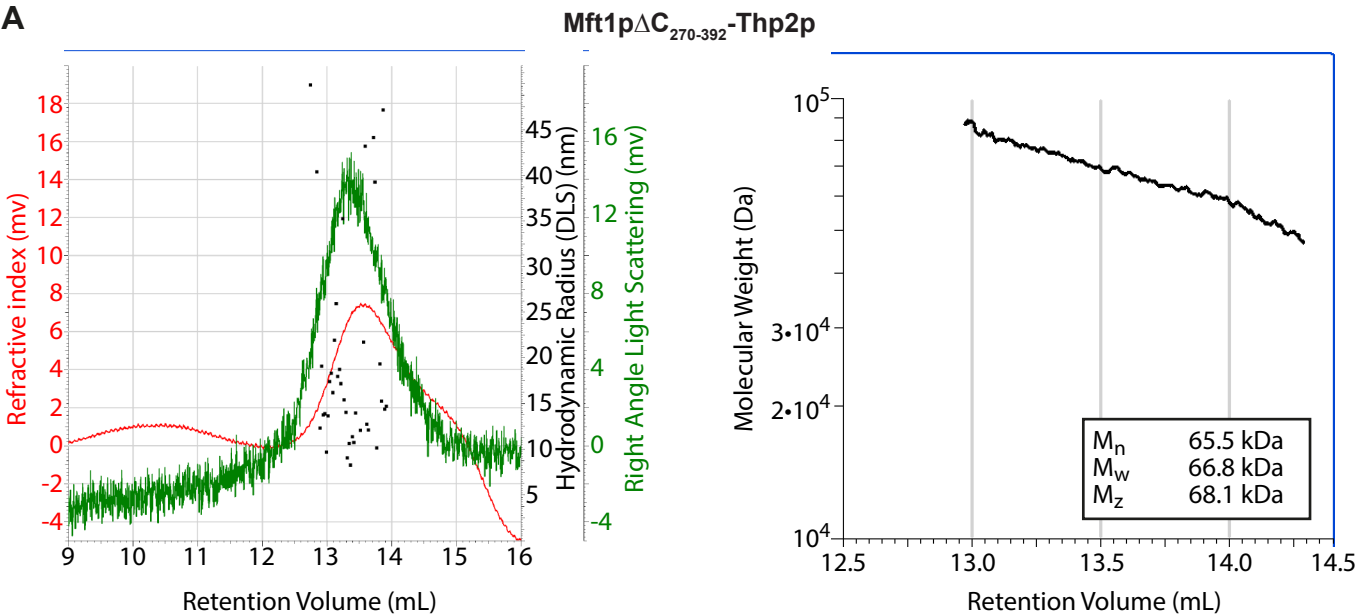

B

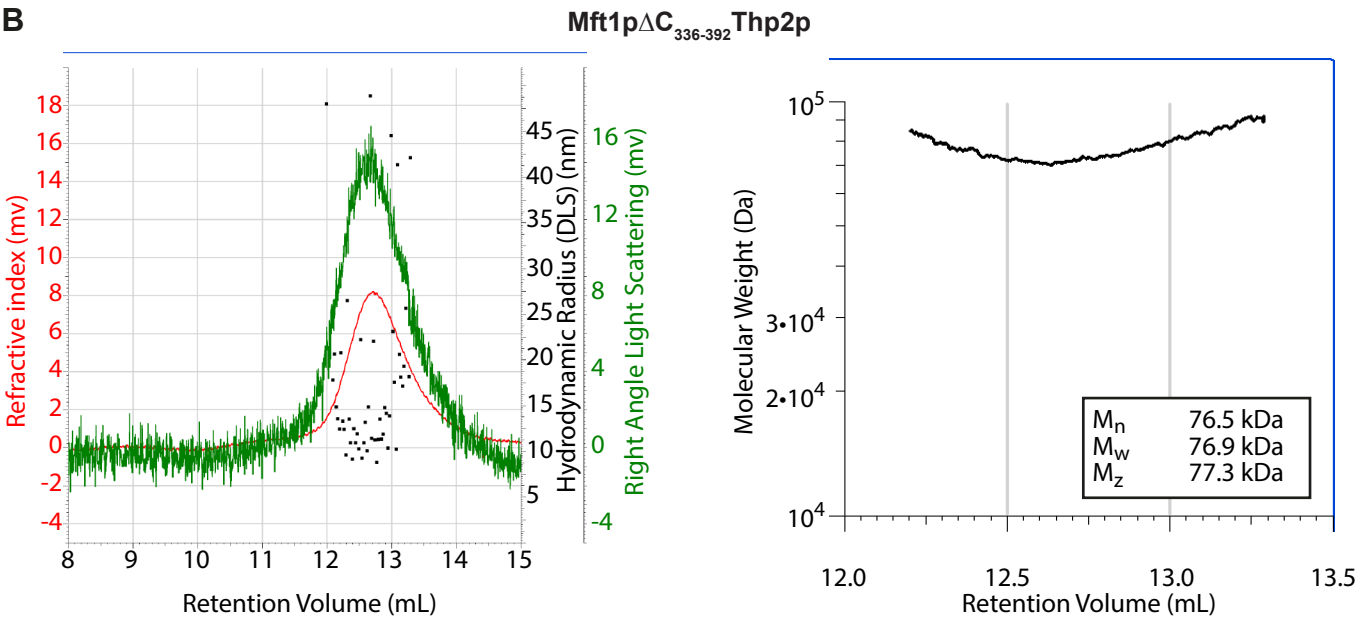

C

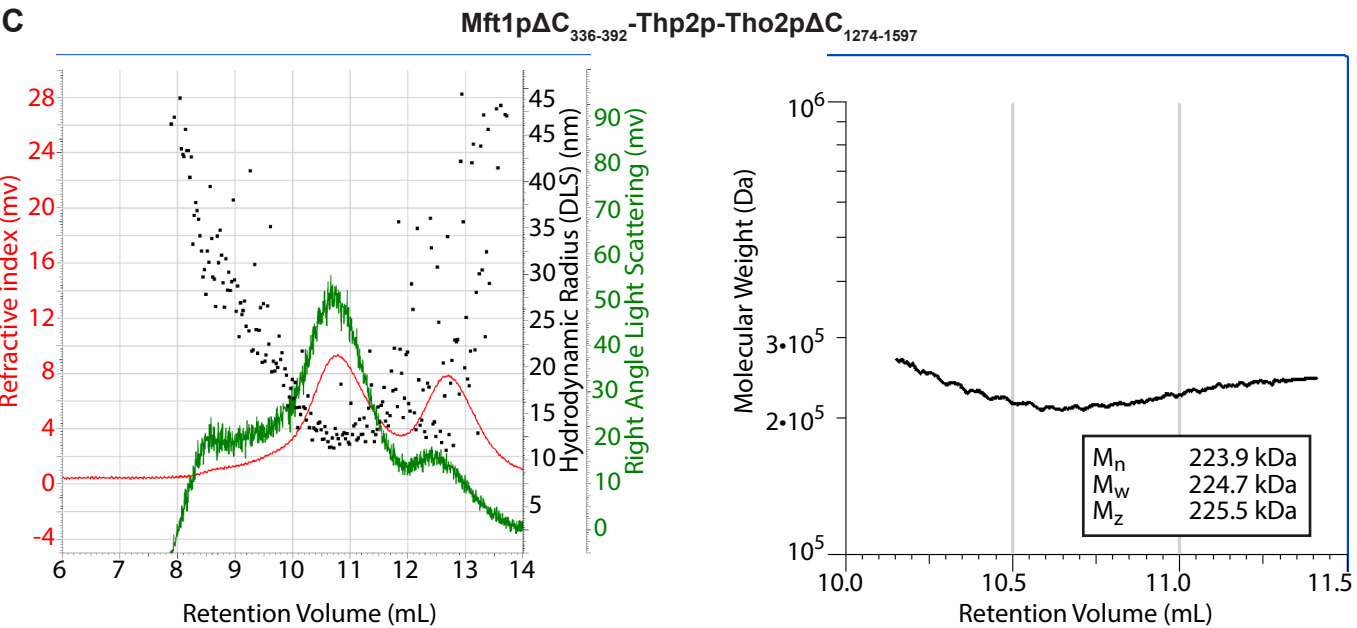

Supplement: Figure S2 — Analysis of complexes by static and dynamic light scattering. A. Mft1pΔC232-392-Thp2p. Left, refractive index (red), measuring protein concentration, right-angle light scattering (RALS) signal (green), and hydrodynamic radius (black dots) as a function of elution volume (mL); Right, absolute molecular weight based on system calibration with bovine serum albumin (BSA), as a function of elution volume. B. As in A, except data for Mft1pΔC336-392-Thp2p. C. As in A, except data for Mft1pΔC336-392-Thp2p-Tho2pΔC1274-1597. (PDF) [file pone.0103470.s002.pdf]

Figure S3

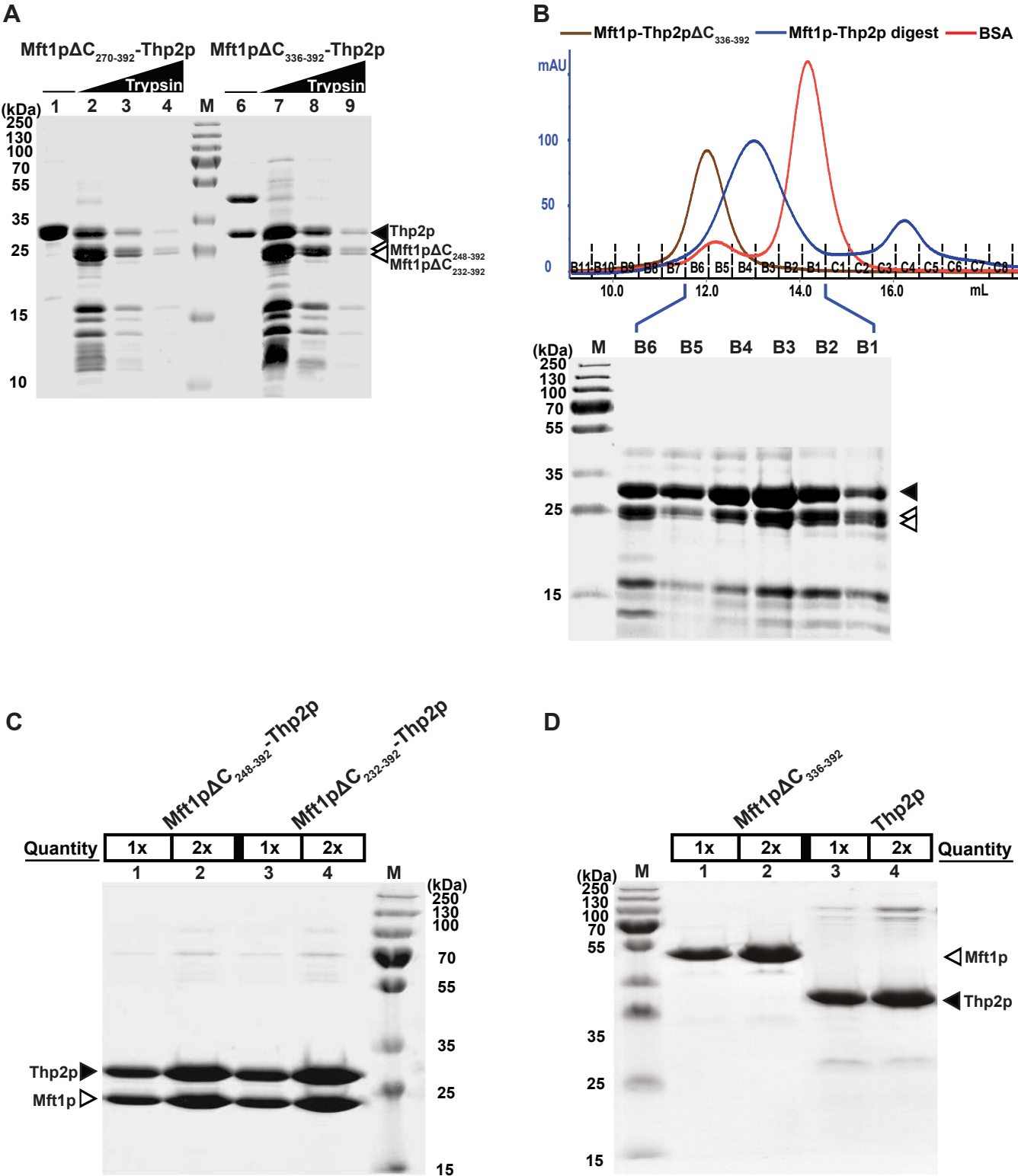

Supplement: Figure S3 — Isolation of truncated subcomplexes of THO by limited proteolysis. A. 20 µg of Mft1pΔC270-392-Thp2p (lanes 1-4) or Mft1pΔC336-392-Thp2p (lanes 6-9) were incubated with increasing concentrations of trypsin for 16 hours at 4°C, and the fractions analysed by Coomassie-stained SDS-PAGE. Lanes 1 and 6: Untreated Mft1p-Thp2p complexes. Arrowheads specify the identified stable protein fragments of Thp2p and Mft1p. M: Marker proteins of indicated molecular weights. B. Mft1pΔC336-392-Thp2p pre-treated with trypsin was analysed on a Superdex 200 HR 10/300 gel-filtration column. The Mft1p-Thp2p digest is shown with a blue line while the intact Mft1pΔC336-392-Thp2p complex is shown with a brown line. The red line is a control sample of bovine serum albumin with a known MW (66 kDa). The y-axis represents mAU absorbance at 280 nm. Note: Both the retention volume and peak area of the digested sample is increased relative to the untreated sample, which suggests formation of several Mft1p-Thp2p complexes of lower overall molecular weight. Below the chromatogram is shown SDS-PAGE analysis (Coomassie-stained) of fractions B6-B1. Arrowheads are as in A and lane M shows marker proteins of known molecular weights. The gel shows protease resistant forms of both proteins. C. Purified samples of binary Mft1pΔC248-392-Thp2p (lanes 1-2) and Mft1pΔC232-392-Thp2p (lanes 3-4) as identified by limited proteolysis. 10 µg (1x) or 20 µg (2x) was loaded in each lane. D. Mft1pΔC336-392 (lanes 1–2) and Thp2p (lanes 3–4) purified in isolation and analysed by Coomassie-stained SDS-PAGE. In both C and D the lane marked M contains marker proteins of known molecular weights as indicated and arrowheads specify the position of indicated proteins. 10 µg (1x) or 20 µg (2x) was loaded in each lane. Protein complexes were purified in four steps: (i) Ni-NTA chromatography, (ii) TEV cleavage and Ni-NTA chromatography, (iii) Q-anion-exchange chromatography and (iv) gel-filtration chromatography, while the isolated p [file pone.0103470.s003.pdf]

Figure S4

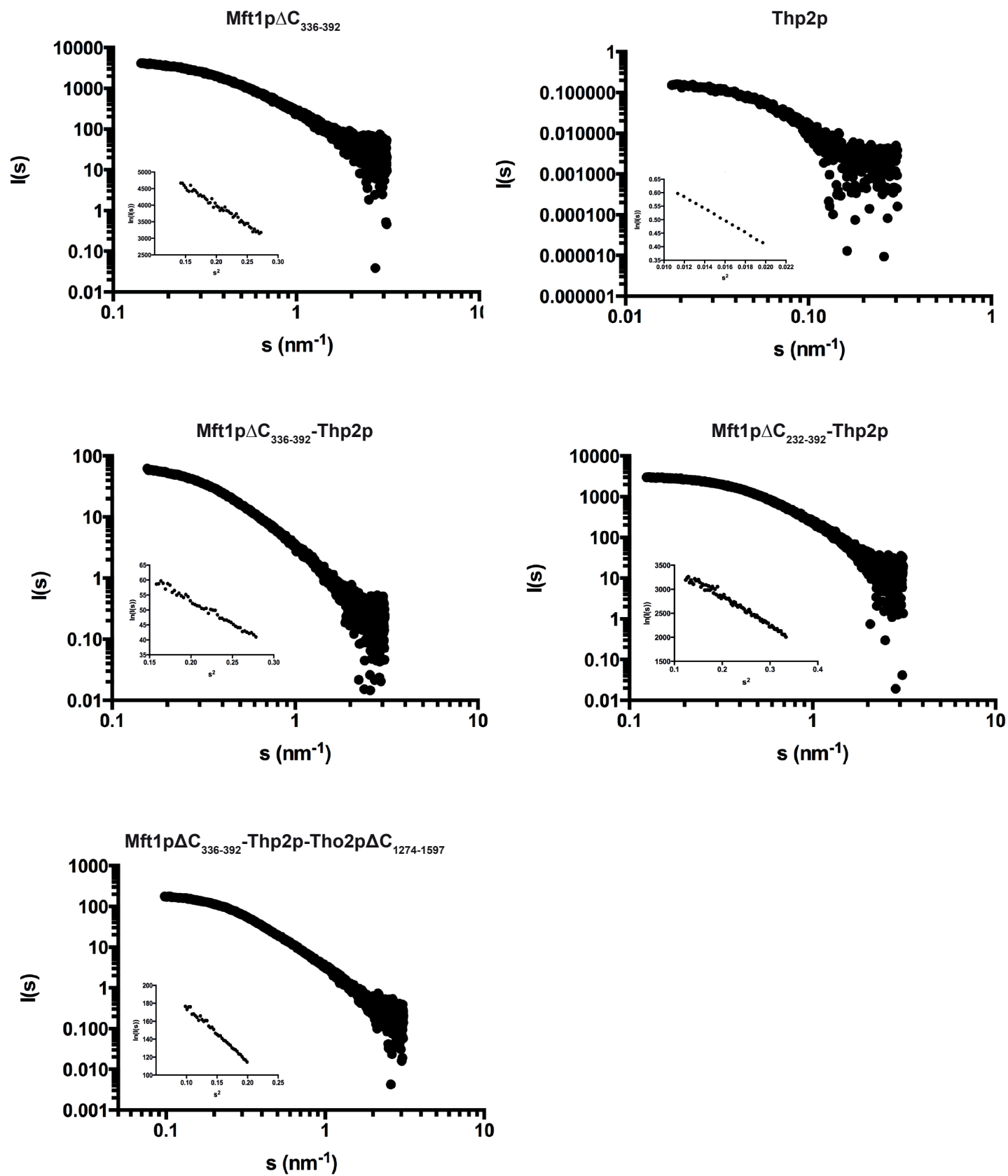

Supplement: Figure S4 — SAXS curves and Guinier plots. SAXS data obtained for each sample as indicated. The Guinier plots (ln(s) versus s2) are inset to show linearity. The corresponding Rg values are shown in Table S1. (PDF) [file pone.0103470.s004.pdf]

Figure S5

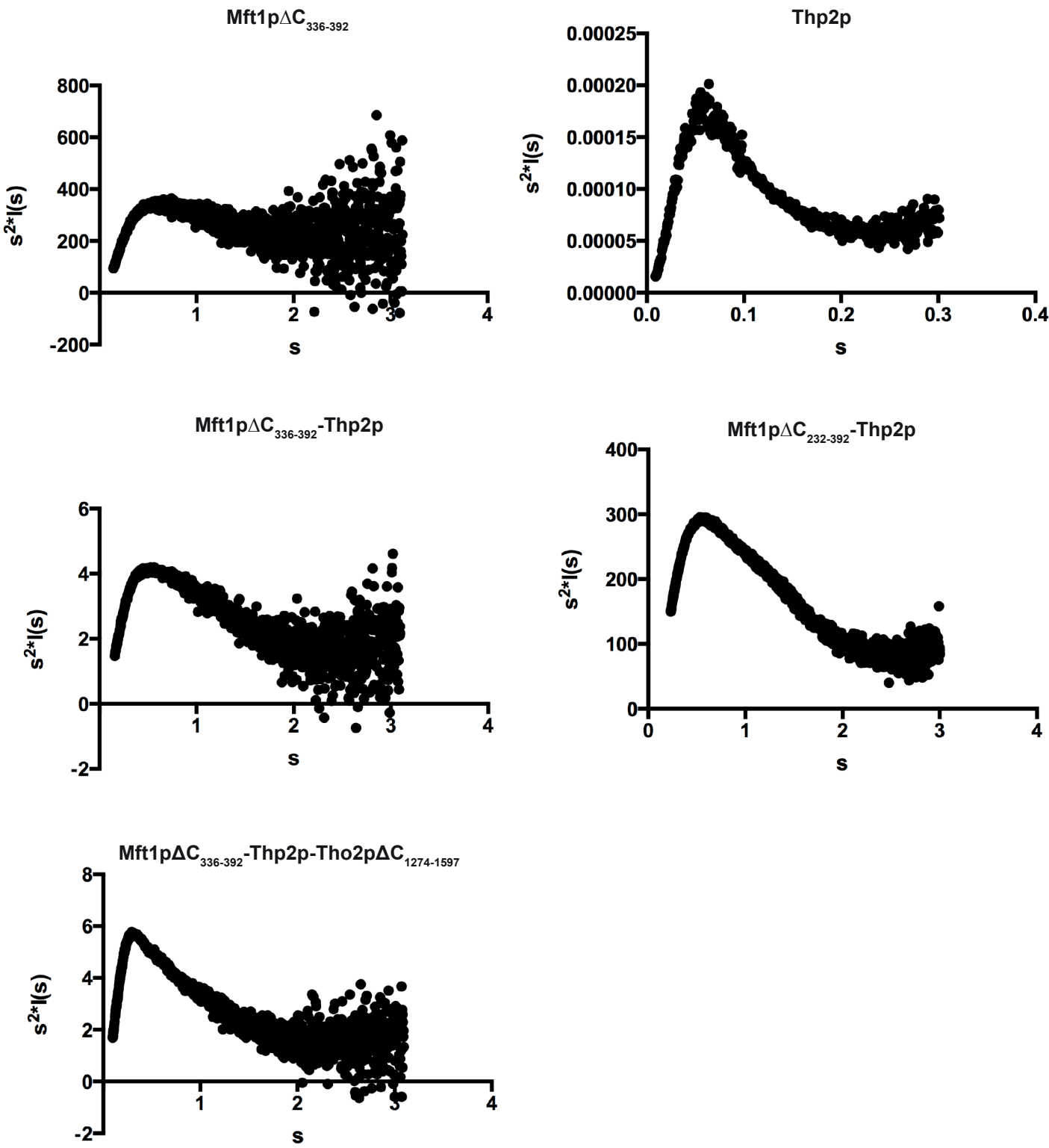

Supplement: Figure S5 — Dimensionless Kratky plots. Kratky plots (s2*l(s) versus s) are shown for each sample as indicated. (PDF) [file pone.0103470.s005.pdf]

Figure S6

Mft1p $\Delta$ C<sub>336-392</sub>

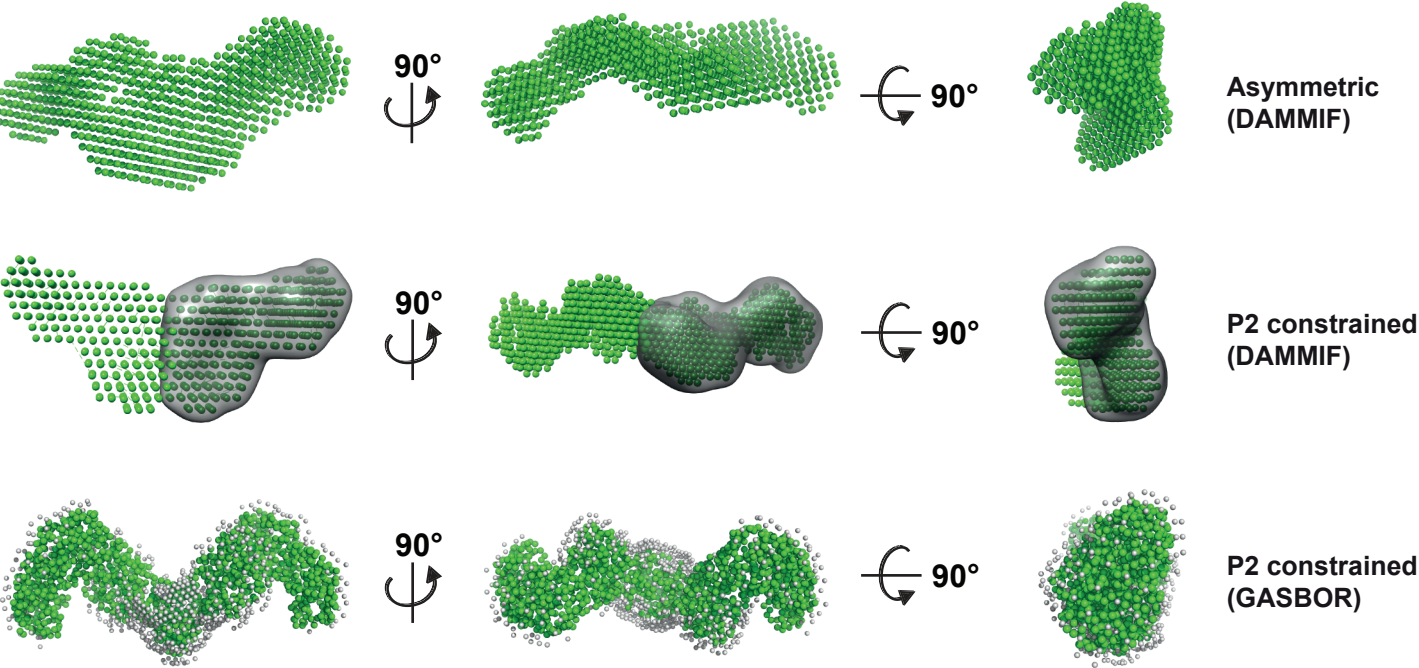

Thp2p

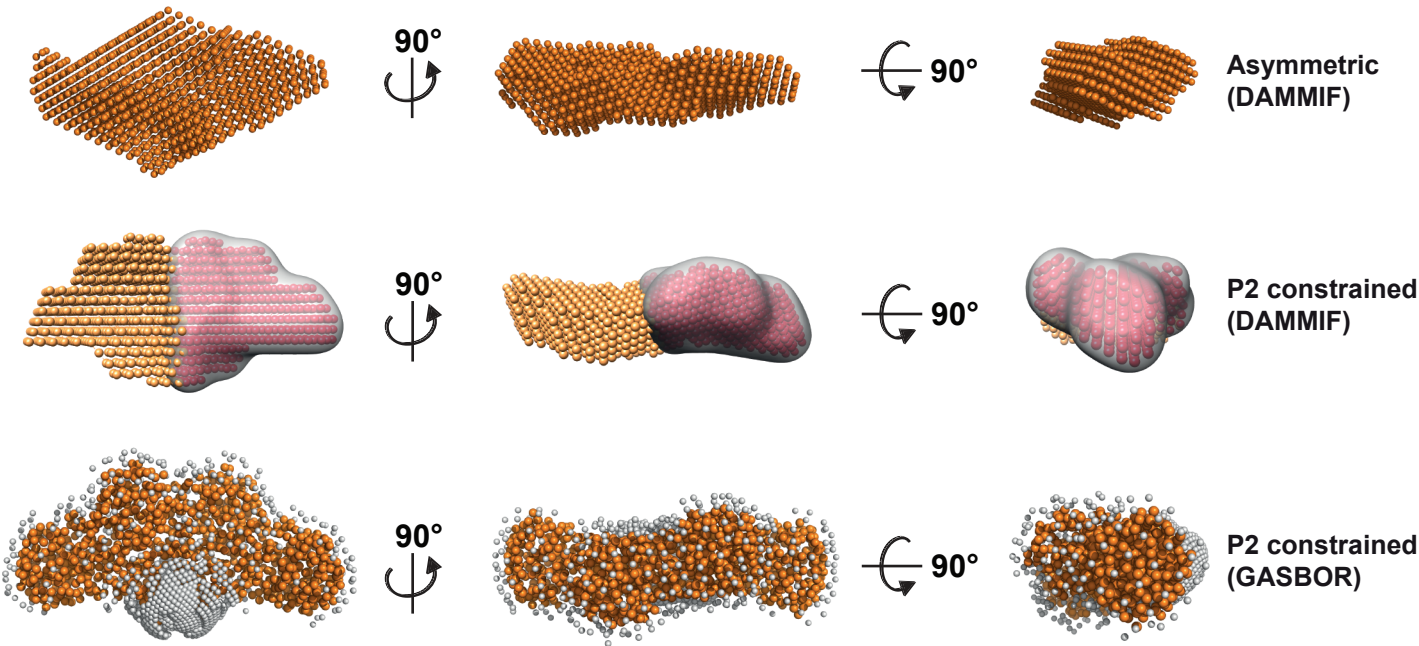

Supplement: Figure S6 — Analysis of Mft1pΔC336-392 and Thp2p homodimers. For each sample (Mft1pΔC336-392, green; Thp2p, orange/red), the asymmetric (non-constrained) and P2-constrained SAXS envelopes calculated using DAMMIF as well as GASBOR are shown in three perpendicular directions, along with the half-volume representing the monomeric protein (semi-transparent surface). (PDF) [file pone.0103470.s006.pdf]

**Figure S7**

**A**

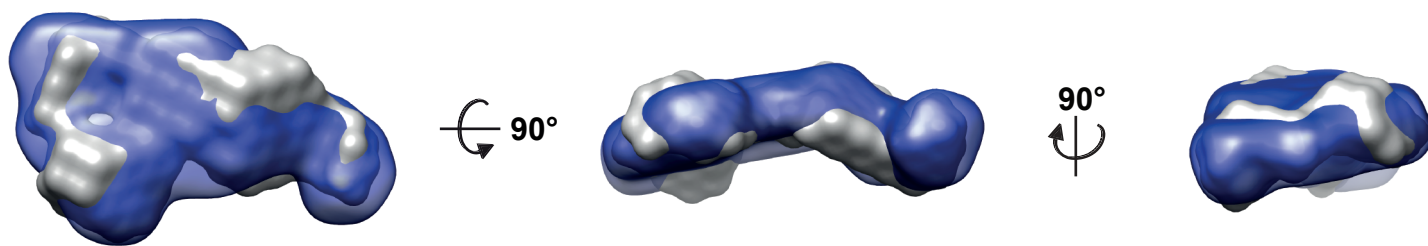

**B**

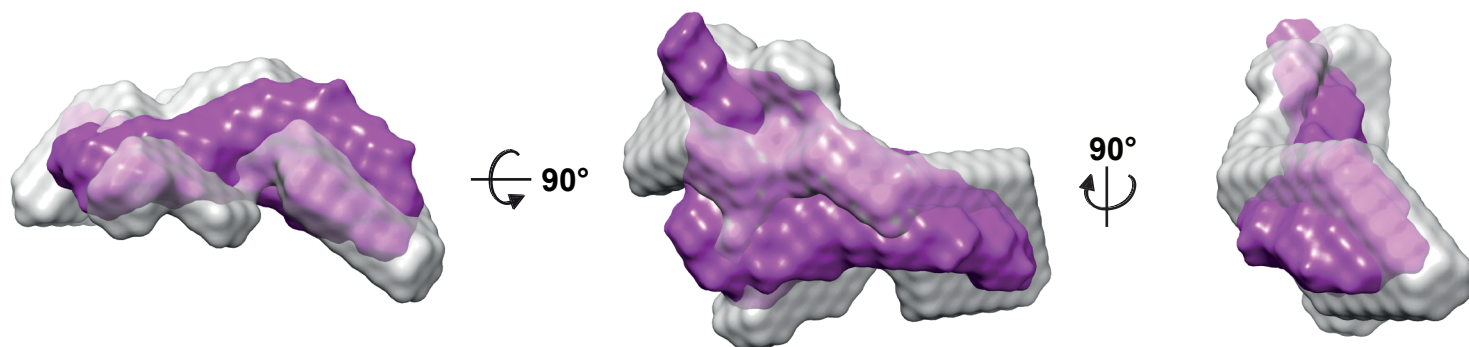

Supplement: Figure S7 — Multiphase-modeling in MONSA. A. Overlay of Mft1pΔC336-392-Thp2p heterodimer representative models from DAMMIF (blue) and MONSA (grey) in three perpendicular views. B. Overlay of Mft1pΔC336-392-Thp2p-Tho2pΔC1274-1597 heterotrimer representative models from DAMMIF (magenta) and MONSA (grey) in three perpendicular views. (PDF) [file pone.0103470.s007.pdf]

Figure S8

A

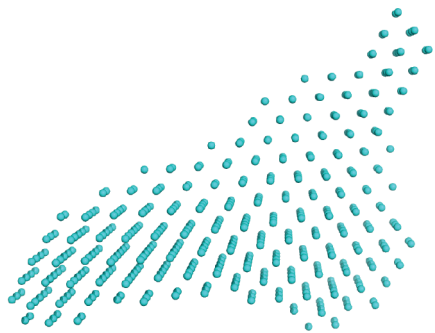

C

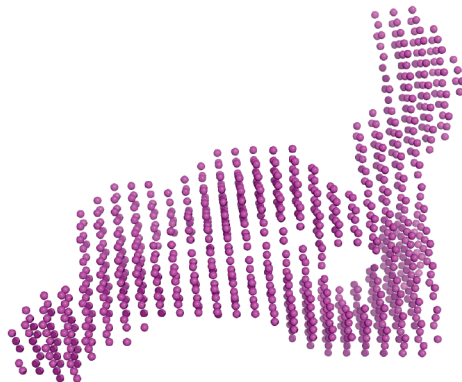

B

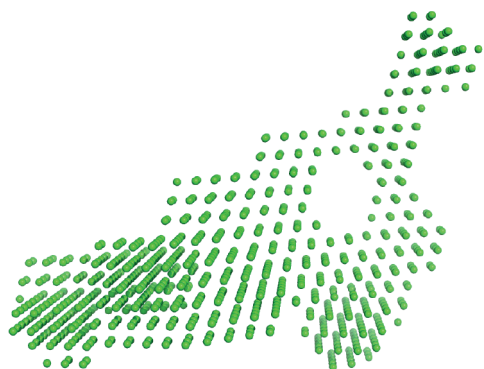

D

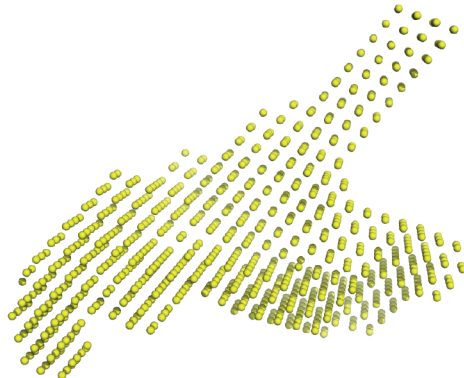

E

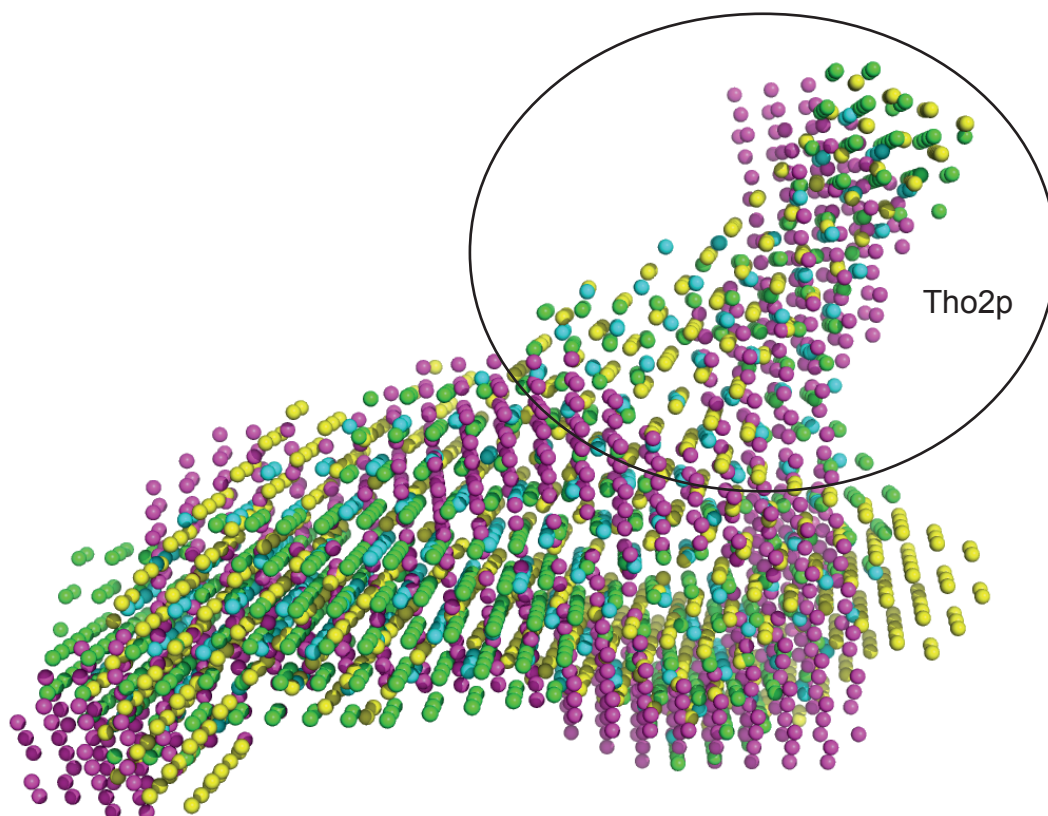

Supplement: Figure S8 — Dummy-atom models based on SAXS data from the trimeric THO complex. A. Filtered average model based on 11 dummy-atom models fitted to the data with a mean Normalised Spatial Discrepancy (NSD) value of 1.108 and variation of 0.056. B. Reference dummy-atom model showing the highest resemblance to the remaining models with an average NSD of 1.028. C. Dummy-atom model showing the second lowest NSD (1.042). D. Dummy-atom model showing the third lowest NSD (1.056). E. Overlay of the models in A-D. Some variation is observed in the part of the trimer corresponding to Tho2p (circled). (PDF) [file pone.0103470.s008.pdf]
