# Supplementary material for: Structural Characterization of the Saccharomyces cerevisiae THO Complex by Small-Angle X-Ray Scattering
Source: PLoS One. 2014 Jul 25;9(7):e103470. doi: 10.1371/journal.pone.0103470 (PMC4111604; doi:10.1371/journal.pone.0103470)
Supplement: Table S1 — Molecular parameters for each sample analysed by SAXS. The table shows the SAXS-derived parameters, Rg, Dmax, and MW. The theoretical MW is the molecular weight calculated based on amino acid sequence and the numbers in parentheses are the corresponding homodimer masses. Experimentally determined MWs were based on comparisons with a BSA standard sample (*based on Porod volume). (PDF) [file pone.0103470.s009.pdf]

Table S1. Molecular parameters for each sample analysed by SAXS

Protein/complex	R_g (nm)	D_{max} (nm)	Measured MW (kDa)	Theoretical MW (kDa)
**Thp2p**	3.4	11.8	68*	33 (66)
**Mft1pΔ$C_{336-392}$**	4.7	16.3	82	40 (80)
**Mft1pΔ$C_{232-392}$-Thp2p**	3.8	13.4	53	59
**Mft1pΔ$C_{336-392}$-Thp2p**	4.6	16.0	71	73
**Mft1pΔ$C_{336-392}$-Thp2p-Tho2pΔ$C_{1274-1597}$**	6.5	22.9	203	220
