# Supplementary material for: Correction: Structural Characterization of the Saccharomyces cerevisiae THO Complex by Small-Angle X-Ray Scattering
Source: PLoS One. 2014 Aug 25;9(8):e106604. doi: 10.1371/journal.pone.0106604 (PMC4143362; doi:10.1371/journal.pone.0106604)
Supplement: File S2 — Republished, corrected article. (PDF) [file pone.0106604.s002.pdf]

OPEN ACCESS Freely available online

PLOS ONE

Structural Characterization of the *Saccharomyces cerevisiae* THO Complex by Small-Angle X-Ray Scattering

Jesper Buchhave Poulsen[1], Lee Edward Sanderson[1], Emil Dandanell Agerschou[1], Emil Dedic[1], Thomas Boesen[2], Ditlev E. Brodersen[1]*

1 Centre for mRNP Biogenesis and Metabolism, Aarhus University, Aarhus, Denmark, **2** Pumpkin - Centre for Membrane Pumps in Cells and Disease, Department of Molecular Biology and Genetics, Aarhus University, Aarhus, Denmark

Abstract

The THO complex participates during eukaryotic mRNA biogenesis in coupling transcription to formation and nuclear export of translation-competent messenger ribonucleoprotein particles. In *Saccharomyces cerevisiae*, THO has been defined as a heteropentamer composed of the Tho2p, Hpr1p, Tex1p, Mft1p, and Thp2p subunits and the overall three-dimensional shape of the complex has been established by negative stain electron microscopy. Here, we use small-angle X-ray scattering measured for isolated THO components (Mft1p and Thp2p) as well as THO subcomplexes (Mft1p-Thp2p and Mft1p-Thp2p-Tho2p) to construct structural building blocks that allow positioning of each subunit within the complex. To accomplish this, the individual envelopes determined for Mft1p and Thp2p are first fitted inside those of the Mft1p-Thp2p and Mft1p-Thp2p-Tho2p complexes. Next, the ternary complex structure is placed in the context of the five-component electron microscopy structure. Our model reveals not only the position of each protein in the THO complex relative to each other, but also shows that the pentamer is likely somewhat larger than what was observed by electron microscopy.

Citation: Poulsen JB, Sanderson LE, Agerschou ED, Dedic E, Boesen T, et al. (2014) Structural Characterization of the *Saccharomyces cerevisiae* THO Complex by Small-Angle X-Ray Scattering. PLoS ONE 9(7): e103470. doi:10.1371/journal.pone.0103470

Editor: Petri Kursula, University of Oulu, Finland

Received December 16, 2013; **Accepted** July 3, 2014; **Published** July 25, 2014

Copyright: © 2014 Poulsen et al. This is an open-access article distributed under the terms of the Creative Commons Attribution License, which permits unrestricted use, distribution, and reproduction in any medium, provided the original author and source are credited.

Funding: This work has been funded by the Danish National Research Foundation's Centre for mRNP Biogenesis and Metabolism and the Lundbeck Foundation. The funders had no role in study design, data collection and analysis, decision to publish, or preparation of the manuscript.

Competing Interests: The authors have declared that no competing interests exist.

* Email: deb@mb.au.dk

Introduction

In eukaryotes, the transcription and processing of pre-mRNAs, formation of messenger ribonucleoprotein particles (mRNPs), and eventually, nuclear export, are tightly linked processes, involving a plethora of protein complexes operating in synergy to deliver translation-competent mRNPs to the cytosol. In essence, these processes are regulated and sustained by numerous transient interactions mediated by protein-protein and protein-nucleic acid contacts, functioning at every step along the way from the site of transcription to the nuclear pore complex (NPC) [1]. Disruption of any of these processes can potentially cause activation of the RNA surveillance machinery and subsequent degradation of mRNAs in the nucleus [2].

One essential component of early mRNA biogenesis is the evolutionarily conserved THO complex, composed in the yeast, *Saccharomyces cerevisiae*, of Tho2p (184 kDa), Hpr1p (88 kDa), Tex1p (47 kDa), Mft1p (45 kDa), and Thp2p (33 kDa). The THO complex accompanies RNA polymerase II during transcription by binding directly to its poly-phosphorylated C-terminal domain (CTD) and facilitates mRNP packaging and export by an unknown mechanism [1,3]. However, it is known that THO co-transcriptionally recruits and binds the mRNA export factors Yra1p (an RNA-binding protein) and Sub2p (an RNA helicase), into a larger assembly that has been termed the *TRanscription and EXport* (TREX) complex [4]. THO also mediates interactions with the poly(A)$^+$ RNA-binding factor, Mex67p, essential for mRNA export, as well as the serine/arginine-rich (SR)-like proteins, Gbp2p and Hrb1p, which stimulate cotranscriptional recruitment of the proteins to nascent mRNA transcripts [4–6]. Furthermore, TREX has been found to transiently interact with the transcription elongation and splicing factor, Prp19p [7].

Depletion or knockout of individual THO complex components *in vivo* has revealed that the complex is not only involved in mRNA biogenesis but also takes part in preserving genome integrity. THO knockout phenotypes usually display decreased levels of nuclear mRNP production leading to stalling of transcription elongation, formation of RNA/DNA hybrid loops (R-loops), genomic instability, and eventually DNA hyper-recombination [8,9]. Deletion of THO components also triggers formation of large aggregates near the nuclear envelope known as *heavy chromatin*, composed of transcriptionally active chromatin, proteins of the RNA export machinery, pre-mRNA, and nuclear pore components [10]. Interestingly, long, GC-rich genes appear to be affected most dramatically in such THO mutant strains [11].

The yeast THO complex was originally characterised as a four-subunit assembly of the proteins Tho2p, Hpr1p, Mft1p, and Thp2p, none of which have known structural domains or functions assigned. However, biochemical and negative-stain electron microscopy (EM) data of complexes purified from native source in *Saccharomyces cerevisiae* suggest that the WD40 repeat protein, Tex1p (47 kDa), is stably associated as well, thus forming a heteropentameric structure [12,13]. Analysis of the THO complex by negative-stain EM has yielded three-dimensional reconstructions of the complex both in the presence and absence of Tex1p

allowing the position of this protein on the surface of the core THO particle to be accurately determined [13]. In addition, the C-termini of Hpr1p and Tho2p were located with the help of an Hpr1p-specific antibody and dynein-tagging of Tho2p [14]. However, the locations of Mft1p, Thp2p, and the N-terminal domains of Tho2p and Hpr1p within the core THO complex have not been described so far.

In this paper, we identify stable subcomplexes of *S. cerevisiae* THO (Mft1p-Thp2p and Mft1p-Thp2p-Tho2p) and use small-angle X-ray scattering (SAXS) to determine envelopes of individual subunits (Mft1p and Thp2p) as well as the subcomplexes. These SAXS envelopes are then used as building blocks for docking all four subunits within the core THO complex. Mft1p, Thp2p, and Tho2p can be positioned with confidence inside the envelope of the ternary Mft1p-Thp2p-Tho2p complex, which is then used for docking into the EM model representing the entire THO core complex [13]. The final model reveals the position of each protein in the complex and further suggests that the overall size of the complex might have been slightly underestimated by the negative-stain EM procedure.

Materials and Methods

Protein expression

For expression screening of multiple THO complexes, bicistronic constructs were prepared encoding Hpr1p-Mft1p and Tho2p-Thp2p, respectively. The Hpr1p-Mft1p constructs were inserted by ligation-independent cloning (LIC) into the pRSF-2 Ek/LIC bacterial expression vector (kanamycin, Novagen) while the Tho2p-Thp2p constructs were inserted into the pET-52b 3C/LIC vector (ampicillin, Novagen) in both cases using the LIC Duet Minimal Adaptor (Novagen). Tho2p and Mft1p contain a primer-encoded C-terminal Strep II fusion tag, Thp2p an N-terminal Strep II fusion tag, and Hpr1p a C-terminal 6xHis-tag. Binary, truncated Mft1p$\Delta C_{232\text{-}392}$-Thp2p and Mft1p$\Delta C_{248\text{-}392}$-Thp2p complexes were inserted as bicistronic constructs into the bacterial pETM-13 expression vector (kanamycin, EMBL) using the NcoI and BamHI restriction sites. A primer-encoded Tobacco Mosaic Virus (TEV) protease-cleavable, C-terminal 8xHis fusion tag was added to Mft1p. Furthermore, Mft1p$\Delta C_{336\text{-}392}$ and Thp2p were LIC-cloned for isolated protein expression into the pET-30 Ek/LIC vector (kanamycin, Novagen), adding a primer-encoded C-terminal 6xHis fusion tag to the protein. All constructs were based on standard *Saccharomyces cerevisiae* genomic DNA as template for PCR (EMD Millipore).

The Hpr1p-Mft1p and Tho2p-Thp2p constructs were co-transformed into *E. coli* Rosetta (DE3) cells and colonies selected on LB agar containing ampicillin, kanamycin, and chloramphenicol, while the Mft1p$\Delta C_{232\text{-}392}$-Thp2p, Mft1p$\Delta C_{248\text{-}392}$-Thp2p, Mft1p$\Delta C_{336\text{-}392}$, and Thp2p constructs were selected using kanamycin and chloramphenicol only. Protein expression was carried out in LB medium with induction at $OD_{600} = 0.8$ with 0.5 mM IPTG and subsequent growth for 18 h at 20°C. Cells were harvested by centrifugation, resuspended in 20 mL of lysis buffer (50 mM Tris-Cl pH 8.0, 300 mM KCl, 5 mM $MgCl_2$, 10% glycerol, and 5 mM 2-mercaptoethanol) per L of cell culture in addition to 1 mM PMSF and 1x Complete Protease Inhibitor tablet (Roche). Cell lysis was achieved by sonication and high pressure homogenization in the presence of 10 μg/mL of DNase I and 40 μg/mL of RNase A. The lysates were finally clarified for purification by ultracentrifugation.

Protein purification

For expression-purification screening of multiple THO complexes, 5% Ni-NTA magnetic agarose beads (Qiagen) were added to clarified lysates (200 μl beads per g of pellet dry weight corresponding to ~200 mL of culture volume) and incubated with rotation at 4°C for 1 h. Samples were washed (50 mM Tris-Cl pH 8.0, 1 M KCl, 5 mM MgCl2, 20 mM imidazole, 10% glycerol, and 5 mM 2-mercaptoethanol) using a magnetic separator and elution (50 mM Tris-Cl pH 8.0, 300 mM KCl, 5 mM MgCl2, 250 mM imidazole, 10% glycerol, and 5 mM 2-mercaptoethanol) was carried out at 4°C for 1 min, after which the eluates were collected as supernatants using a magnetic separator. Elution fractions were combined with 10% Strep-Tactin magnetic beads (200 μl per g of pellet dry weight) (Qiagen) and incubated with rotation at 4°C for 1 h. Proteins were washed again (50 mM Tris-Cl pH 8.0, 300 mM KCl, 5 mM MgCl2, 10% glycerol, and 5 mM 2-mercaptoethanol) and eluted (50 mM Tris-Cl pH 8.0, 300 mM KCl, 5 mM MgCl2, 10% glycerol, 10 mM biotin, and 5 mM 2-mercaptoethanol) using the magnetic separator. For purification of the Mft1pΔC336-392-Thp2p, Mft1pΔC336-392-Thp2p-Tho2pΔC1274-1597 and Mft1pΔC270-392-Thp2p complexes, clarified lysates were loaded onto a Strep-Tactin Superflow Plus Cartridge column (Qiagen) pre-equilibrated in lysis buffer. Unbound protein was removed with lysis buffer, and bound proteins eluted (50 mM Tris-Cl pH 8.0, 300 mM KCl, 5 mM MgCl2, 2.5 mM d-desthiobiotin 10% glycerol, and 5 mM 2-mercaptoethanol). Elution fractions were diluted four-fold to 50 mM Tris-Cl pH 8.0, 75 mM KCl, 5 mM MgCl2, 2.5 mM d-desthiobiotin, 2.5% glycerol, and 5 mM 2-mercaptoethanol and applied to a Source 15Q column pre-equilibrated in (50 mM Tris-Cl pH 8.0, 100 mM KCl, 5 mM MgCl2 and 5 mM 2-mercaptoethanol). Elution (50 mM Tris-Cl pH 8.0, 1 M KCl, 5 mM MgCl2, and 5 mM 2-mercaptoethanol) was carried out with a gradient extending from 0-100% over 30 mL. Fractions containing the purified complexes were identified by Coomassie-stained SDS-PAGE gels, then pooled and concentrated using a Vivaspin concentrator (Sartorius). Protein complexes were finally purified using size-exclusion chromatography running in 50 mM Tris-Cl pH 8.0, 100 mM KCl, 5 mM MgCl2, and 5 mM 2-mercaptoethanol using a Superdex 200 GL 10/300 column (GE Healthcare). Subsequent rounds of size-exclusion chromatography (SEC) were carried out to separate ternary Mft1pΔC336-392-Thp2p-Tho2pΔC1274-1597 from binary Mft1pΔC336-392-Thp2p formed following expression of the complex THO3. A total of ~2 mg (Mft1pΔC336-392-Thp2p), ~0.1 mg (Mft1pΔC336-392-Thp2p-Tho2pΔC1274-1597) and ~3 mg (Mft1pΔC270-392-Thp2p) of purified complexes could be obtained per L of cell culture. To purify Mft1pΔC232-392-Thp2p and Mft1pΔC248-392-Thp2p, the clarified lysates were loaded onto a Ni-NTA Superflow Cartridge column (Qiagen) equilibrated in lysis buffer. Unbound proteins were washed out (50 mM Tris-Cl pH 8.0, 300 mM KCl, 5 mM MgCl2, 20 mM imidazole, 10% glycerol, and 5 mM 2-mercaptoethanol), and the bound fractions eluted (50 mM Tris-Cl pH 8.0, 300 mM KCl, 5 mM MgCl2, 250 mM imidazole, 10% glycerol, and 5 mM 2-mercaptoethanol). Elution fractions treated with His-tagged recombinant TEV protease using a ratio of 1:100 (protease: total protein by mass) and dialyzed overnight against lysis buffer. TEV-cleaved samples were re-applied to the Ni-NTA cartridge and the flow-through collected. Flow-through fractions containing target Mft1p-Thp2p complexes were then diluted four-fold to 50 mM Tris-Cl pH 8.0, 75 mM KCl, 5 mM MgCl2, 2.5% glycerol, and 5 mM 2-mercaptoethanol and further purified using the Source 15Q and Superdex 200 GL 10/300 columns (GE Healthcare) as

described above. ~2 mg (Mft1pΔC232-392-Thp2p) and ~3 mg (Mft1pΔC248-392-Thp2p) of purified complexes were obtained per L of cell culture. Isolated Mft1pΔ$C_{336\text{-}392}$ and Thp2p proteins were purified as for Mft1pΔ$C_{232\text{-}392}$-Thp2p and Mft1pΔ$C_{248\text{-}392}$-Thp2p, respectively, however without TEV cleavage and the 2nd Ni-NTA step. ~1 mg of each protein was obtained per L of cell culture.

Biochemistry

Purified proteins/complexes were concentrated for biochemical analysis using Vivaspin concentrators and characterized in regard to size and molecular weight using a Zetasizer μV instrument (Malvern) connected inline to the Superdex 200 GL 10/300 column for both static (SLS) and dynamic (DLS) light scattering measurements. Proteins were identified by MALDI-TOF-MS of peptides derived by in-gel trypsin (Sigma) digestion of excised gel spots. *In situ* proteolysis was carried using serially diluted trypsin for 16 h at 4°C, in which the degradation fragments were N and C terminally characterized by Edman degradation and MALDI-TOF-MS analyses, respectively. Protein complexes were designed and sub-cloned based on this information. Comparison of relative protein amounts by densitometric analyses was carried out using the Image Quant TL software (GE healthcare).

Small-angle X-ray scattering

Synchrotron radiation SAXS data were collected at the EMBL SAXS-WAXS beam line X33 at DORIS/DESY (Hamburg, Germany), the MAX-lab I911-4 SAXS beamline and the P12 beamline EMBL SAXS-WAXS at PETRAIII/DESY. The X33 data were collected as 8x 15 sec exposures at 1.5 Å using a MAR345 image plate detector, and scattering profiles for the 8 passes were compared to detect radiation damage. The I911-4 data were collected as 2x 30 sec exposures at 1.5 Å using a Pilatus detector, while the P12 data were collected as 20x 0.05 sec exposures and scattering profiles for the 20 passes were compared to detect radiation damage. Measurements were conducted at 10°C for X33 and P12 and 25°C for I911-4 using 10-75 μL sample in 50 mM Tris-Cl pH 8.0, 100 mM KCl, 5 mM MgCl2, and 5 mM 2-mercaptoethanol. Measurements were carried out at 3-6 different concentrations in all cases using concentrations between 7.8 mg/ml and 0.15 mg/ml. Background scattering was subtracted using ATSAS [15] and PRIMUS [16]. Linear Guinier plots in the Guinier region ($s*R_g<1.3$) were confirmed in all cases (Figure S4). Pair distance distribution functions of the particles P(r) and the maximum sizes D_{max} were computed using GNOM [17] and molecular weights were estimated by comparison of the extrapolated forward scattering I(0) of the samples obtained using Guinier analysis by AUTORG [18] with that of a bovine serum albumin standard (Sigma-Aldrich) (Table S1). Porod volumes were calculated using ATSAS AUTOPOROD [18] and *ab initio* shapes were determined using DAMMIF [19], in the case of the Thp2p and Mft1p homodimers, both with and without a P2 symmetry constraint (Figure S6). After 12 DAMMIF runs, DAMAVER [20] was used to analyse the normalized spatial discrepancy (NSD) between the 12 models and the lowest NSD model was used as representative, except for the heterotrimeric complex where the filtered model was used. Envelopes were calculated using SITUS pdb2vol based on the DAMMIF models [21]. The model envelopes were docked manually in UCSF Chimera and map correlation coefficients were determined [22]. Enantiomer versions of all envelopes were tested for optimal fit. For the Mft1p and Thp2p homodimers, GASBOR modeling was also carried out using P2 symmetry for comparison (Figure S6) [23]. Multiphase *ab initio* modeling using both the Mft1pΔ$C_{336\text{-}392}$-Thp2p heterodimer and Mft1pΔ$C_{336\text{-}392}$-Thp2p-Tho2pΔ$C_{1274\text{-}1597}$ trimer data was carried out using the online version of MONSA (http://www.embl-hamburg.de/biosaxs/atsas-online/) [24]. The measured R_g value of 4.6 nm for the heterodimer (Table S1), a spherical search volume with a radius of 11.5 nm, and P1 symmetry was imposed during this refinement where a total of 12 runs were compared.

Results

Isolation of binary and ternary subcomplexes of THO

Initially, a His-tagged version of the intact, tetrameric *Saccharomyces cerevisiae* THO complex consisting of the proteins Tho2p, Hpr1p, Mft1p, and Thp2p was expressed and purified from *E. coli* Rosetta (DE3) using two compatible plasmids each encoding two full-length proteins (Hpr1p-Mft1p in pRSF-2 Ek/LIC and Tho2p-Thp2p in pET52b 3C/LIC). However, using this setup, expression yields were quite low and Tho2p and Hpr1p showed signs of significant degradation. To stabilize the proteins during expression, we introduced serial N and C terminal truncations in Tho2p and Hpr1p. Since no detailed structural information is available for any of the THO proteins, the truncations were based on sequence analysis and avoiding interruption of predicted secondary structure elements. Two variants of Mft1p were also generated with C terminal deletions of 5 and 13 kDa, respectively. In total, the truncations yielded eight plasmids encoding bicistronic Hpr1p-Mft1p constructs and four plasmids encoding bicistronic Tho2p-Thp2p constructs (Figure 1A and S1A). Tho2p, Mft1p, and Thp2p were expressed as Strep II Tag fusion proteins while Hpr1p contained a 6xHis-Tag fusion, allowing for two-step affinity purification. The plasmids were combined by co-transformation to yield 32 unique combinations of the THO complex that were tested for expression level and stability. A complete overview of the constructs and combinations prepared can be found in Figure S1.

All THO complex variants were screened and ranked in terms of expression yield and stability of the individual proteins in order to identify those most suitable for structural studies. Briefly, complexes were expressed in *E. coli* Rosetta (DE3) cells, purified by a two-step affinity purification protocol involving Ni-NTA magnetic agarose beads followed by Strep-Tactin magnetic beads, and finally, eluted and analysed by Coomassie Blue-stained SDS-PAGE. Figure 1B (left hand side) shows the results for a subset of the complexes, THO1, THO3, and THO4 (see figure legend for details). Stable forms of Mft1p and Thp2p were observed in nearly all cases, whereas neither Tho2p nor Hpr1p could be identified in these experiments. Mft1p and Thp2p were present in near equal stoichiometric amounts as determined by densitometric analysis of the gels (data not shown) suggesting formation of a two-component subcomplex of THO consisting of the two proteins. This hypothesis was further supported by the detection of Mft1p and Thp2p in the Strep-Tactin eluate fraction of THO3 versus their absence for complex THO2 for which Mft1p lacks the Strep II tag (Figure S1B). In essence, the analysis showed that only the Strep II Tag fusion of Mft1p is functional and thus that Thp2p appears in the Strep-Tactin elution fraction due to interaction with Mft1p, not due to its own Strep II tag fusion, which in this case seems to be occluded. Although Mft1p and Thp2p are the only proteins purified in these experiments, Hpr1p and Tho2p could still be expressed and present substoichiometrically. To examine this possibility, the purification was repeated using large-scale expression cultures (Figure 1B, right). Interestingly, this yielded a band for THO3 migrating at the expected size for Tho2p, which was confirmed by mass spectrometry. This band corresponds to a C-terminally truncated version of the Tho2p protein, Tho2pΔ$C_{1274\text{-}1597}$ (Figure 1B and S1C). App. 2 mg of the Mft1pΔ$C_{336\text{-}392}$-

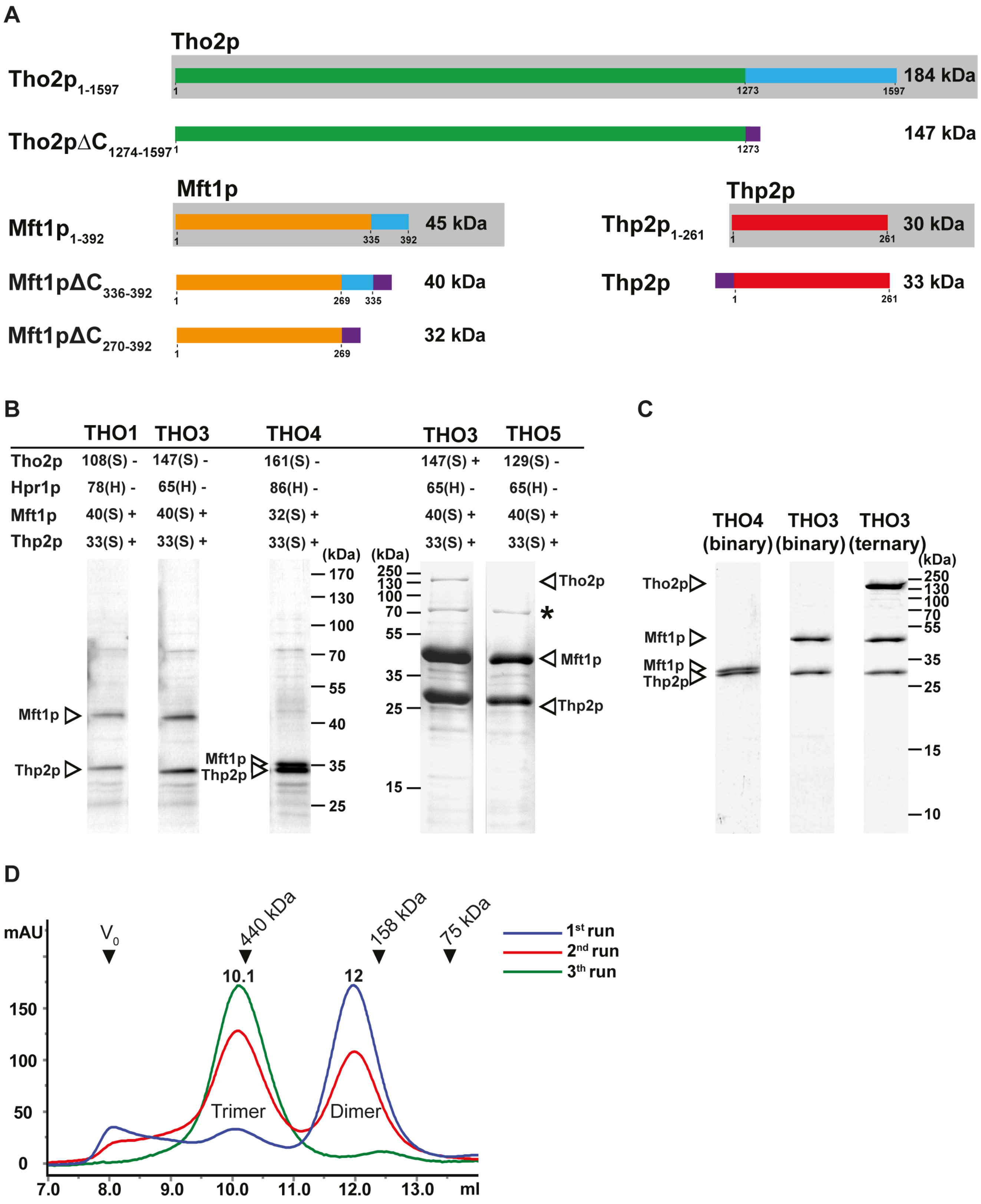

Figure 1. Purification of THO subcomplexes. A. Overview of the Tho2p, Mft1p and Thp2p constructs used relative to their full-length forms (boxed in grey). Blue boxes: Stretches of residues removed to obtain the construct directly below. Purple boxes: Strep II tag. **B.** THO complexes (THO1, 3, 4, and 5) were expressed and purified from *E. coli* in two steps and analysed by Coomassie-stained SDS-PAGE. The table shows for each construct the molecular weight of each protein and any associated tags (H = 6xHis, S = Strep II) as well as whether the protein is expressed (+/-). Positions on the gel for proteins confirmed by mass spectrometry are indicated with arrowheads and * indicates an *E. coli* protein contaminant. **C.** Purified THO subcomplexes analysed by Coomassie-stained SDS-PAGE: Heterodimeric THO4 (Mft1pΔ$C_{270\text{-}392}$-Thp2p), heterodimeric THO3

(Mft1pΔ$C_{336\text{-}392}$-Thp2p), and heterotrimeric THO3 (Mft1pΔ$C_{336\text{-}392}$-Thp2p-Tho2pΔ$C_{1274\text{-}1597}$). **D.** Overlay of gel filtration chromatograms obtained during isolation of the ternary Mft1pΔ$C_{336\text{-}392}$-Thp2p-Tho2pΔ$C_{1274\text{-}1597}$ (Trimer) from the binary Mft1pΔ$C_{336\text{-}392}$-Thp2p (Dimer). Between runs 1 (blue), 2, (red), and 3 (green), peak fractions were pooled, concentrated and re-applied to the column. Elution retention volumes are noted along with the positions of standards used for calibration: Blue Dextran (V_0, void volume, 2000 kDa), ferritin (440 kDa), aldolase (158 kDa), and conalbumin (75 kDa). Units on the y-axis are mAU absorption at 280 nm.
doi:10.1371/journal.pone.0103470.g001

Thp2p and ~0.1 mg of the Mft1pΔ$C_{336\text{-}392}$-Thp2p-Tho2pΔ$C_{1274\text{-}1597}$ complex could be purified per L of expression cell culture using strain THO3. In conclusion, we thus find that binary complexes consisting of Mft1p and Thp2p most likely result following expression of THO3 and THO4, i.e. Mft1pΔ$C_{336\text{-}392}$-Thp2p and Mft1pΔ$C_{270\text{-}392}$-Thp2p, respectively. Furthermore, the expression pattern observed for complex THO3 indicates that a subcomplex of Mft1p, Thp2p, and Tho2p could have been formed as well.

Characterisation of binary and ternary subcomplexes of THO

To identify and characterize the observed subcomplexes of THO, the strains expressing THO3 and THO4 were grown in larger cultures and purified using a three-step procedure involving Strep-Tactin chromatography, anion-exchange chromatography, and gel filtration chromatography. During these experiments, we were able to isolate both binary (Mft1pΔ$C_{336\text{-}392}$-Thp2p) and ternary (Mft1pΔ$C_{336\text{-}392}$-Thp2p-Tho2pΔ$C_{1274\text{-}1597}$) THO subcomplexes from THO3, whereas a binary subcomplex (Mft1pΔ$C_{270\text{-}392}$-Thp2p), containing a more truncated copy of Mft1p, could be isolated following expression of THO4 (Figure 1C). Using successive rounds of gel filtration chromatography it was further possible to separate the ternary and binary forms expressed in THO3 (Figure 1D). The purified complexes were analysed by densitometric analysis of the SDS-PAGE gels, which suggested that they contain the proteins in equal stoichiometric amounts as expected (data not shown). Furthermore, molecular masses of 60-70 kDa (Mft1pΔ$C_{270\text{-}392}$-Thp2p) 70-80 kDa (Mft1pΔ$C_{336\text{-}392}$-Thp2p), and 220-230 kDa (Mft1pΔ$C_{336\text{-}392}$-Thp2p-Tho2pΔ$C_{1274\text{-}1597}$) were estimated for the subcomplexes using static light scattering (Figure S2), which is in good agreement with the calculated, theoretical molecular masses of 65, 73, and 220 kDa, respectively.

Structural analysis of THO subcomplexes

To identify further stable, truncated complexes of binary Mft1pΔ$C_{270\text{-}392}$-Thp2p and Mft1pΔ$C_{336\text{-}392}$-Thp2p we used limited proteolysis followed by sub-cloning and expression. An overview of the proteolysis experiments leading to isolation of the truncated subcomplexes Mft1pΔ$C_{232\text{-}392}$-Thp2p and Mft1pΔ$C_{248\text{-}392}$-Thp2p as well as the purification of these subcomplexes is shown in Figure S3. We next analysed the Mft1pΔ$C_{336\text{-}392}$-Thp2p binary complex and the more truncated Mft1pΔ$C_{232\text{-}392}$-Thp2p form identified by limited proteolysis as well as the Mft1pΔ$C_{336\text{-}392}$-Thp2p-Tho2pΔ$C_{1274\text{-}1597}$ ternary complex by small-angle X-ray scattering (SAXS). In addition, isolated Mft1pΔ$C_{336\text{-}392}$ and full length Thp2p were purified and analysed (Figure S3D). SAXS data were collected at beam lines Doris X33 (DESY, Hamburg), Petra P12 (DESY, Hamburg) and I911-4 (MAX-lab, Sweden) and data quality evaluated using Guinier plots (Figure S4). Dimensionless Kratky plots displayed the characteristic peak expected for folded proteins for all samples (Figure S5). The SAXS data was first used to calculate the maximum particle dimension (D_{max}) and radius of gyration (R_g) for each sample, which showed highly similar values at all evaluated concentrations for each of the complexes (Table S1). For isolated Mft1pΔ$C_{336\text{-}392}$ and Thp2p, inter-particle effects were observed at high concentrations and measurements at low concentrations suggested that they both form stable homodimers in solution (Table S1).

The experimental SAXS data were next used for the reconstruction of twelve individual *ab initio* molecular envelopes using dummy bead modelling in the program DAMMIF. For each protein or complex, the most representative model was picked as the one having the lowest Normalised Spatial Discrepancy (NSD) compared to the rest of the models (Figure 2). Envelopes calculated without symmetry constraints for both Mft1pΔ$C_{336\text{-}392}$ and Thp2p were elongated and exhibited quasi two-fold symmetry around their short axes, suggesting each half corresponded to a monomer of the homodimeric molecules (Figure S6). To generate the envelope corresponding to the isolated monomeric molecules, we applied P2 symmetry constraints in DAMMIF and isolated the half-volumes corresponding to the monomers. These monomer volumes were output in the resulting DAMMIF representative model PDB files as the symmetry-related bead volumes and assessed for a compact globular fold as a basic assumption for correct monomer assignment (Figure S6). We also carried out modelling of the Mft1p and Thp2p homodimers using the program GASBOR, which consistently showed overall very similar structures (Figure S6). Both the D_{max} values and the derived envelopes demonstrate that Mft1p is more elongated than Thp2p, which on the other hand seems to adopt a more globular form (Figure 2A and B). Compared to the symmetrical homodimers, envelopes calculated from the binary Mft1pΔ$C_{232\text{-}392}$-Thp2p and Mft1pΔ$C_{336\text{-}392}$-Thp2p complexes consistently showed asymmetric envelopes consisting of a bulky and a thin end (Figures 2C and D). Furthermore, the model calculated for the more truncated version of the binary complex, Mft1pΔ$C_{232\text{-}392}$-Thp2p, showed a smaller overall Porod volume (140 nm^3) than the more intact complex, Mft1pΔ$C_{336\text{-}392}$-Thp2p (194 nm^3). Finally, the ternary Mft1pΔ$C_{336\text{-}392}$-Thp2p-Tho2pΔ$C_{1274\text{-}1597}$ complex exhibited a substantially larger volume, triangular in shape with one flat and broad surface at the base and a narrow protrusion at the top (Figure 2E). For all complexes, the dimensions of the averaged envelopes were consistent with the experimentally determined R_g and D_{max} values even though the DAMMIF modelling allows expansion of the search volume in the simulated annealing step (Table S1).

Placement of individual subunits in the THO complex

Next, we manually docked the SAXS envelopes of Mft1p and Thp2p into the envelopes obtained for the larger binary and ternary complexes to determine their relative position (Figure 3). Initially, docking of the envelope of the smaller binary Mft1pΔ$C_{232\text{-}392}$-Thp2p complex into the larger Mft1pΔ$C_{336\text{-}392}$-Thp2p model resulted in a very good fit (map correlation of 0.933) with additional density in the thin end of the model to account for the shorter C-terminus of Mft1p in the smaller complex (Figure 3A). In fact, the difference envelope volume closely correlates with the expected size difference of ~15 kDa compared to the larger Mft1pΔ$C_{336\text{-}392}$-Thp2p envelope, suggesting it represents the C terminus of Mft1p (red arrow in Figure 3A). Next, we docked the corresponding monomer envelopes of isolated Mft1p and Thp2p into the two binary complex envelopes

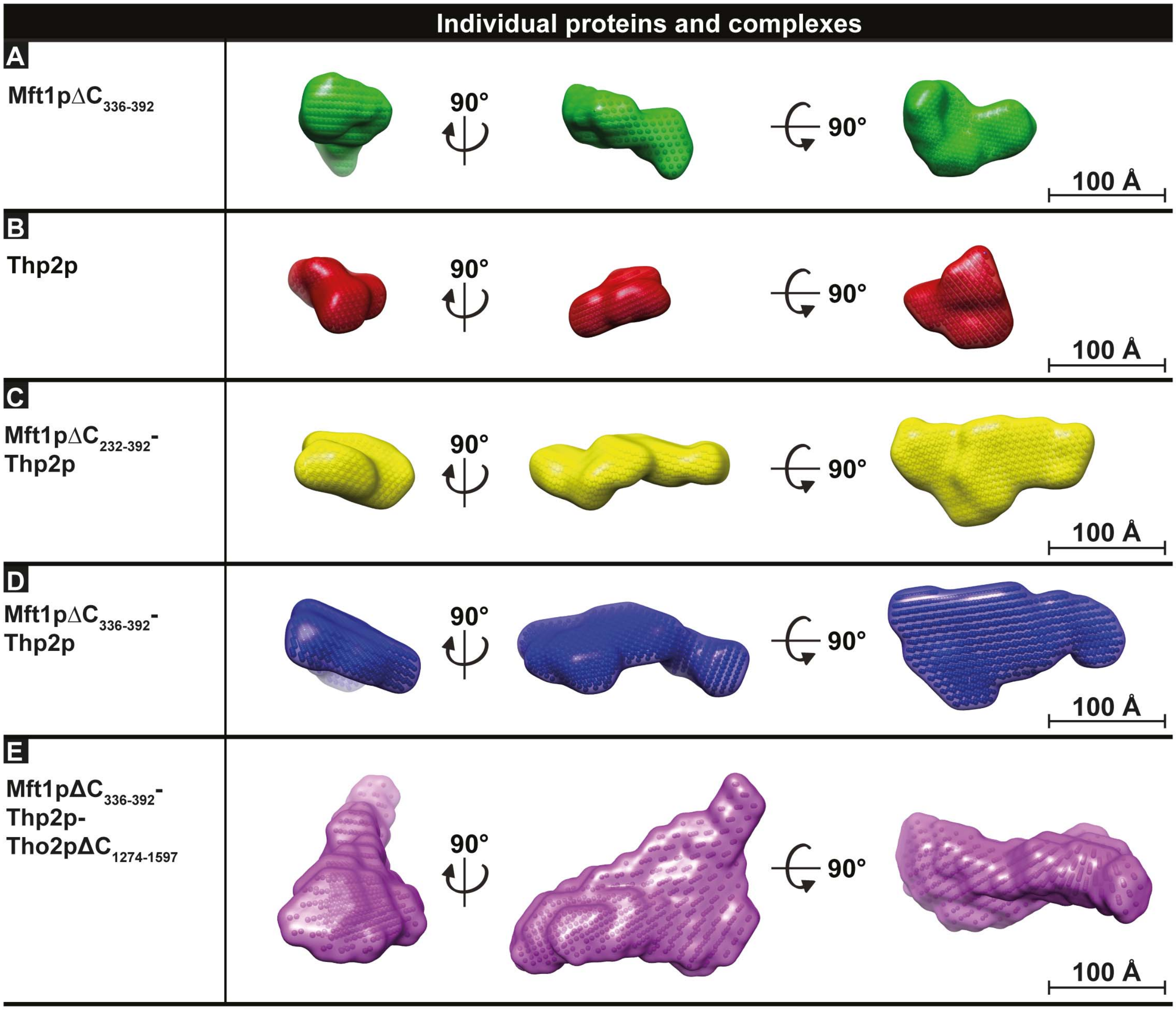

Figure 2. Envelopes of THO proteins and complexes obtained by SAXS. Molecular envelopes obtained using DAMMIF for THO proteins and subcomplexes. **A**. Mft1pΔ$C_{336\text{-}392}$ (as isolated from the homodimer). **B**. Thp2p (as isolated from the homodimer). **C**. Mft1pΔ$C_{232\text{-}392}$-Thp2p. **D**. Mft1pΔ$C_{336\text{-}392}$-Thp2p. **E**. Mft1pΔ$C_{336\text{-}392}$-Thp2p-Tho2pΔ$C_{1274\text{-}1597}$. The proteins and complexes are each represented with an envelope shown in three perpendicular views as indicated. Scale bars represent 100 Å.
doi:10.1371/journal.pone.0103470.g002

to assess the position of each subunit (map correlation 0.903 and 0.931, respectively, Figures 3B and C). In this docking, we placed the thinner end of Mft1p into the thin end of the binary complex envelope, consistent with the shape of the isolated Mft1p protein as well as the truncation results described above. In addition, we assume that Thp2p and Mft1p dimerise in isolation due to the lack of a binding partner, thus, we have oriented the two proteins in the heterodimer in such a way that they interact using their dimerisation interfaces.

In the published model of the THO complex based on negative stain EM, the C-terminal region of the large Tho2p protein was located at a narrow protrusion extending away from the core complex [13]. Our SAXS model of the ternary Mft1pΔ$C_{336\text{-}392}$-Thp2p-Tho2pΔ$C_{1274\text{-}1597}$ complex also contains such a protrusion, suggesting that this may represent the C-terminal region of Tho2p as well (red arrow in Figure 3D). In addition, the envelopes calculated for the binary THO complexes fit nicely along the "floor" of the remaining ternary envelope (map correlation of 0.909), essentially allowing us to place all subunits in the trimer with good confidence (Figure 3D). Figure 4A summarises the proposed position and orientation of Mft1p, Thp2p and Tho2p inside the SAXS envelope of the trimer as supported by our data, with the orientation of Mft1p being supported by our truncation data. This model suggests that Tho2p could interact with both Mft1p or Thp2p. It also suggests that the interaction are likely facilitated by elements in the N-terminal region of Tho2p as the C-terminal region is located in the protrusion. Likewise, Mft1p is predicted to interact with Thp2p through its N-terminal region based on our truncation experiments.

To corroborate this model, *ab initio* multi-phase models were prepared based on the Mft1pΔ$C_{336\text{-}392}$-Thp2p (dimer) and Mft1pΔ$C_{336\text{-}392}$-Thp2p-Tho2pΔ$C_{1274\text{-}1597}$ (trimer) data using MONSA [24]. In this procedure, models of both heterodimer, trimer, and the isolated Tho2pΔ$C_{1274\text{-}1597}$ subunit as well as their

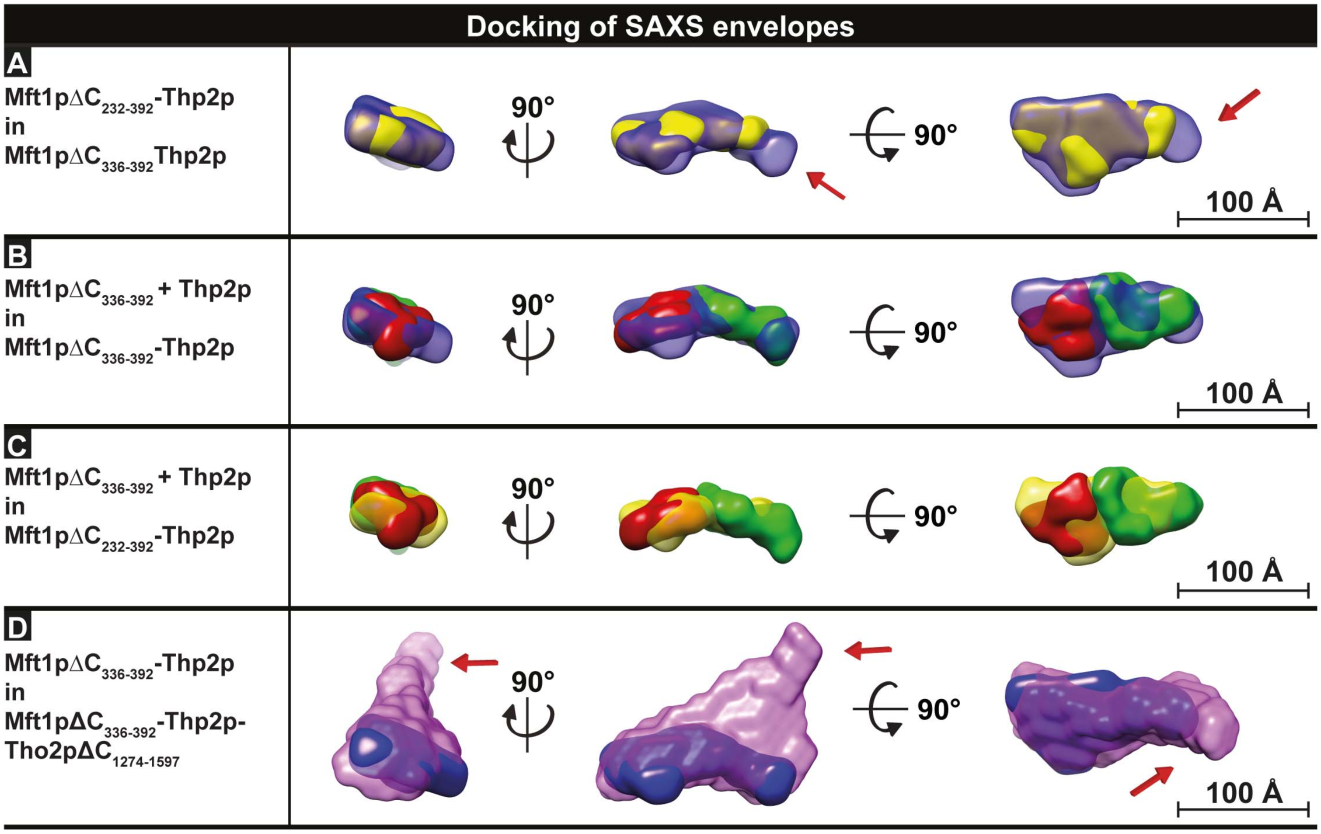

Figure 3. Docking of THO substructures. A. Docking of the Mft1pΔ$C_{232-392}$-Thp2p heterodimer (yellow) into the larger Mft1pΔ$C_{336-392}$-Thp2p heterodimer (blue). The arrows indicate the proposed position of the Mft1p C-terminus. **B.** Docking of isolated Mft1pΔ$C_{336-392}$ (green) and Thp2p (red) into the larger, heterodimeric Mft1pΔ$C_{336-392}$-Thp2p envelope (blue). **C.** Docking of isolated Mft1pΔ$C_{336-392}$ (green) and Thp2p (red) into the smaller, heterodimeric Mft1pΔ$C_{232-392}$-Thp2p envelope (yellow). **D.** Docking of the larger, dimeric Mft1pΔ$C_{336-392}$-Thp2p envelope (blue) into the ternary Mft1pΔ$C_{336-392}$-Thp2p-Tho2pΔ$C_{1274-1597}$ envelope (purple). The arrows indicate the proposed position of the Tho2p C-terminus. Scale bars represent 100 Å.
doi:10.1371/journal.pone.0103470.g003

relative orientations are produced by including both data sets in a single analysis. The heterodimer models derived from MONSA displayed a low mean value of NSDs of 0.682 and the resulting models overlap very well with those derived from DAMMIF (Figure S7A). The mean value of NSDs for the trimer models derived from MONSA is 0.907, which is relatively high but again similar to the trimer model produced by DAMMIF (1.028, Figure S7B). Consequently, the model representing the part of the molecule attributed to Tho2pΔ$C_{1274-1597}$ also displayed a high mean value of NSDs of 0.857 and thus showed more variability compared to the Mft1-Thp2 heterodimer. However, in both cases, the models had similar dimensions and shapes to those derived from DAMMIF.

Finally, we manually docked the SAXS model of the ternary THO complex into the structure of the five-component THO complex determined by negative stained EM, taking into account the already known positions of Tex1p, Hpr1p, and the Tho2p C-terminus (Figure 4B) [13]. Initial comparison of the envelope volumes suggested that our SAXS model has a greater volume for Mft1p, Thp2p and Tho2p than the EM reconstruction (data not shown). Whereas a good fit could be obtained for both Mft1p and Thp2p by positioning them at the base of the croissant-like structure extending towards Hpr1p in the EM model, Tho2p was found to occupy a significantly larger volume in the SAXS envelope than in the EM reconstruction. This may, in part, be due to flexibility as the top-three dummy-atom models based on the Normalised Spatial Discrepancy values from on the pairwise comparison of 11 calculated models show some variation in this region (Figure S8). However, the fit of the Thp2p-Mft1p part of the SAXS envelope is good and furthermore, the region corresponding to Hpr1p in the EM reconstruction is not covered by the SAXS envelope as well, consistent with the absence of this protein from our samples. In summary, by combining our SAXS data with the available EM reconstruction, we have been able to produce a model that describes the position of all subunits of the pentameric THO complex (Figure 5). In addition, we have provided structural evidence that the C-terminal half of Mft1p is located in proximity to Hpr1p, while the N-terminal half is nearer Thp2p. This architectural model for the pentameric THO complex should provide a solid basis for design of functional and genetic studies to elucidate the function of the individual subunits during mRNP biogenesis.

Discussion

The THO complex is a central nuclear component involved in mRNP formation by linking transcription with export of translation-competent mRNAs by an unknown mechanism. The yeast THO complex is a heteropentameric protein assembly consisting of Tho2p, Hpr1p, Tex1p, Mft1p, and Thp2p, capable of interacting with a plethora of proteins, amongst others Yra1 and Sub2 forming the TREX complex [4,5,13]. Recently, part of the

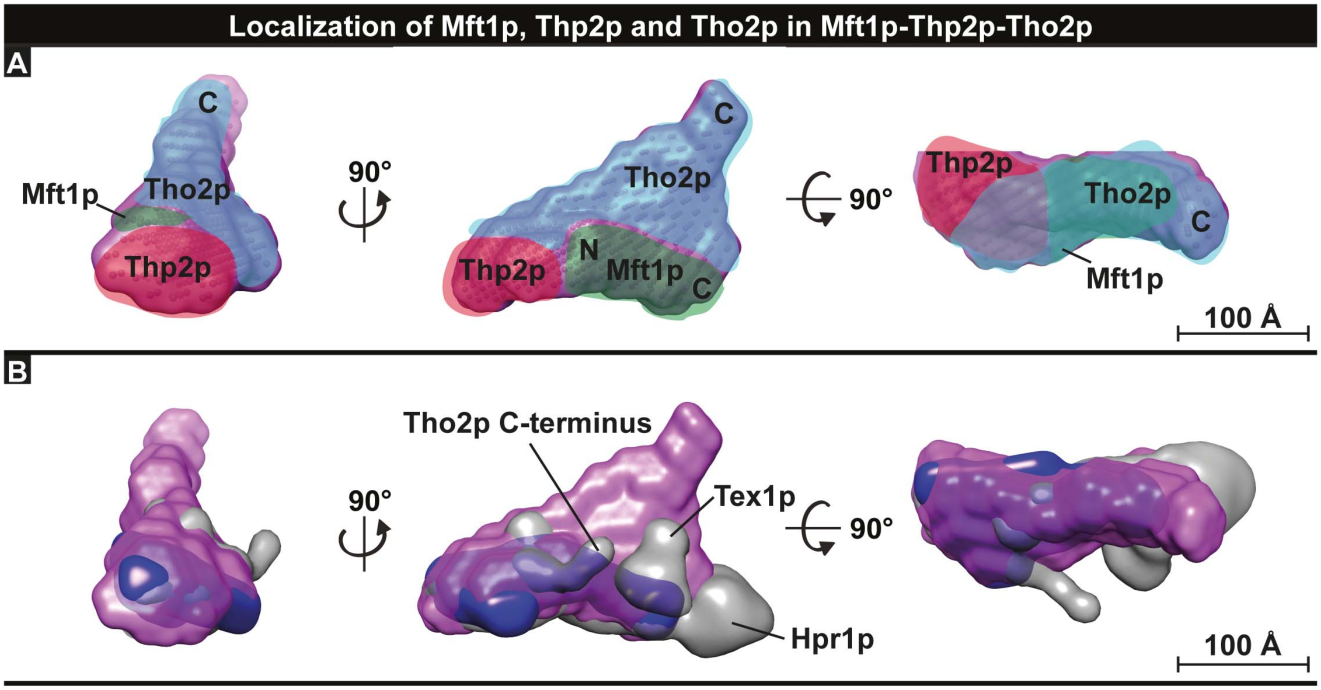

Figure 4. Placement of subunits in the THO complex. A. Model showing the proposed location and orientation of the subunits Mft1p (green), Thp2p (red), and Tho2p (blue) within the ternary Mft1p$\Delta C_{336\text{-}392}$-Thp2p-Tho2p$\Delta C_{1274\text{-}1597}$ THO complex (purple). The proposed location of the N and C terminal regions of Tho2p and Mft1p are indicated. Scale bars represent 100 Å. **B.** Comparison of the ternary Mft1p$\Delta C_{336\text{-}392}$-Thp2p-Tho2p$\Delta C_{1274\text{-}1597}$ SAXS envelope with the five-component EM reconstruction of the THO complex [13]. The positions of the proteins not part of our sample (Hpr1p and Tex1p) are indicated.
doi:10.1371/journal.pone.0103470.g004

architecture of the *Saccharomyces cerevisiae* THO complex was established by negative stain EM that was used to generate a three-dimensional reconstruction of the molecule [13]. The model was described as a croissant-like structure with a flat surface at the base and two large protrusions, one long and thin and the other shorter but wider. The EM model allowed placement of Tex1p and Hpr1p, as well as the Tho2p C-terminus using a range of techniques including mutational studies, immunochemistry, and biochemistry. Despite this effort, the positions of Mft1p, Thp2p, and the Tho2p N-terminus within the complex remained unclear.

In this study, we have used small-angle X-ray scattering to determine envelope structures for individual THO components and subcomplexes, which allow us to uniquely position each subunit in the THO complex. Internally, all SAXS envelopes are consistent and can be docked into the larger subcomplexes with relative ease. A good fit was obtained for both Mft1p and Thp2p along the base of the croissant-like shape determined by EM,

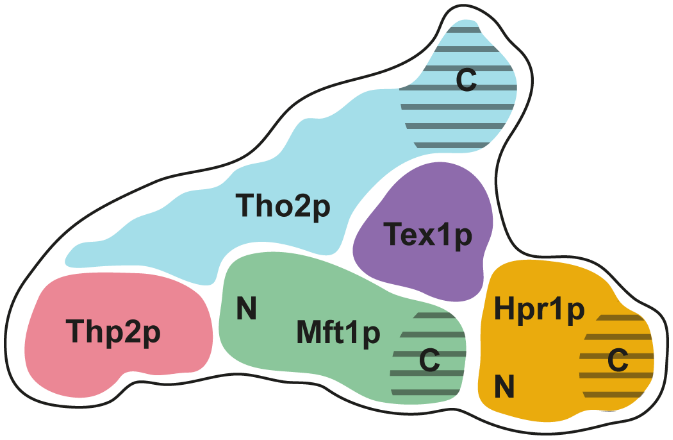

Figure 5. Overview of the composition of the THO complex. Model showing the proposed architectural arrangement of subunits within the intact, pentameric THO complex based on the combined EM and SAXS data. The positions and N/C terminal orientation of Thp2p and Mft1p are based on this study, while the positions and orientation of Hpr1p, Tex1p, and Tho2p are mainly based on [13]. Hatched areas indicated predicted flexible regions.
doi:10.1371/journal.pone.0103470.g005

however, Tho2p was found to occupy a larger volume than expected from the EM reconstruction. We believe there may be several reasons for this discrepancy. Firstly, this region of the THO complex may be flexible, causing it to appear larger in the SAXS envelope and smaller in the EM model due to the averaging of particles. Supporting this interpretation is the fact that Pena *et al.* determined three-dimensional EM reconstructions of a series of truncated THO complexes with varying lengths of the Tho2p C-terminus without observing any differences between the reconstructions [13]. This is despite the deletions comprising a considerable part of the protein with the largest corresponding to removal of residues 1274-1597 of Tho2p or more than 35 kDa. Consequently, the outermost C-terminal parts of Tho2p (~320 residues) are unlikely to be visible in the published EM reconstruction. This might either be due to flexibility of this region and consequently that it assumes different structures in the particles used for averaging during EM reconstruction, or as a result of the negative staining process.

Pena *et al.* also provided the first mechanistic insights into how the THO complex may be recruited to co-transcriptionally active genes by showing that the highly basic C-terminus of Tho2p (residues 1279-1597) allows chromatin recruitment through binding of nucleic acids [13]. However, since the association of the THO complex with active chromatin was not completely abolished upon C-terminal deletion of Tho2p, it was also proposed that other components of the THO complex might contain exposed, flexible regions that could be involved in DNA binding. Recently, it has been shown that THO interacts directly with RNA polymerase II through its poly-phosphorylated C-terminal domain (CTD) [3]. To further assess whether disordered regions might be present in the other components of THO, we performed a search for disordered regions with the GlobPlot 2.3 prediction server using the full-length sequences of Mft1p, Thp2p, Tex1p, and Hpr1p [25]. In this search we identified the following C-terminal regions as potentially disordered: Mft1p, residues 330–396 (63 residues, ~7 kDa, pI 4.01); Hpr1p, residues 703–752 (50 residues, ~5 kDa, pI 3.85); Thp2p, residues 246–261 (16 residues, ~2 kDa, pI 3.83), and Tex1p, residues 388–422 (35 residues, ~4 kDa, pI 12.02). The disordered region of Tex1p, which is highly basic (pI 12.01) is adjacent to the likewise basic C-terminus of Tho2p in the model. We therefore speculate that the C-termini of Tex1p and Tho2p may work in conjunction to form a hybrid domain structure of highly positively charged residues, possibly important for chromatin and/or RNA binding. It is also possible that this positive patch interacts directly with the RNA polymerase II CTD through negatively charged, phosphorylated serine residues [3]. The disordered regions of Mft1p, Hpr1p, and Thp2p are, in contrast, acidic with pI values of 4.01, 3.85, and 3.83, respectively. In fact, negatively charged, disordered regions occur more frequently than regions of positively charged nature in nuclear proteins, and these regions have been proposed to take part in transient protein-protein interactions by means of low-affinity interactions [26-28]. The C-terminal regions of Mft1p and Hpr1p may therefore constitute general interaction platforms (or scaffolds) required for protein-protein interactions during mRNP maturation. Consistent with this idea, recruitment of the export factors Sub2p and Mex67p to the THO complex during mRNP export is known to be facilitated through interaction with the C-terminus of Hpr1p, in which the Hpr1p-Mex67p interaction is dependent on ubiquitination of Hpr1p [29-31]. Also, deletion of the Hpr1p C-terminus results in severe phenotypes including impaired mRNP formation and genomic instability, together suggesting an important role for the Hpr1p C-terminus in mRNA biogenesis [32]. In conclusion, we have in this paper completed the architectural description of THO by localising each protein in the complex. Our model reveals the orientation and position of each subunit in the complex and provides new clues to explain the mechanistic details of this assembly. Further experimental evidence will now be needed to understand the molecular mechanisms underlying recruitment of the THO complex to active chromatin during early mRNA biogenesis as well as to determine the detailed three-dimensional structure of the complex.

Supporting Information

Figure S1 Overview of constructs, truncations and combinations of the THO complex**. A.** Overview of Tho2p, Hpr1p, Mft1p, and Thp2p truncation variants. The proteins were named according to their full-length form (grey boxes). Blue boxes: stretch of residues removed in the construct directly below. Purple boxes: Strep II Tag. Red boxes: 6xHis-Tag. Expected molecular weights based on sequence are indicated. Inset: Combination of two plasmids during co-transformation yields a total of 32 combinations of the THO complex. Proteins and genes were coloured as in A. **B.** 10 µg (1x) or 20 µg (2x) of final eluates of THO1-4 as analysed by Coomassie-stained SDS-PAGE (lanes 1–8). Lane 9: Marker proteins of indicated molecular weights. **C.** 20–100 µg of final eluates of THO3 and THO5 analysed by Coomassie-stained SDS-PAGE (lanes 2–7). Lane 1: Marker proteins of indicated molecular weights. In both B and C, the expected protein molecular weight (MW), the fusion tag (H: 6xHis-Tag, S: Strep II Tag) and whether that protein is expressed (+/-) are indicated. Arrowheads: black fill, Thp2p; white fill, Mft1p; gradient, Tho2p; asterisk, a protein contaminant.
(PDF)

Figure S2 Analysis of complexes by static and dynamic light scattering. **A.** Mft1p$\Delta C_{232\text{-}392}$-Thp2p. Left, refractive index (red), measuring protein concentration, right-angle light scattering (RALS) signal (green), and hydrodynamic radius (black dots) as a function of elution volume (mL); Right, absolute molecular weight based on system calibration with bovine serum albumin (BSA), as a function of elution volume. **B.** As in A, except data for Mft1p$\Delta C_{336\text{-}392}$-Thp2p. **C.** As in A, except data for Mft1p$\Delta_{C336\text{-}392}$-Thp2p-Tho2p$\Delta C_{1274\text{-}1597}$.
(PDF)

Figure S3 Isolation of truncated subcomplexes of THO by limited proteolysis. **A.** 20 µg of Mft1p$\Delta C_{270\text{-}392}$-Thp2p (lanes 1-4) or Mft1p$\Delta C_{336\text{-}392}$-Thp2p (lanes 6-9) were incubated with increasing concentrations of trypsin for 16 hours at 4°C, and the fractions analysed by Coomassie-stained SDS-PAGE. Lanes 1 and 6: Untreated Mft1p-Thp2p complexes. Arrowheads specify the identified stable protein fragments of Thp2p and Mft1p. M: Marker proteins of indicated molecular weights. **B.** Mft1p$\Delta C_{336\text{-}392}$-Thp2p pre-treated with trypsin was analysed on a Superdex 200 HR 10/300 gel-filtration column. The Mft1p-Thp2p digest is shown with a blue line while the intact Mft1p$\Delta C_{336\text{-}392}$-Thp2p complex is shown with a brown line. The red line is a control sample of bovine serum albumin with a known MW (66 kDa). The y-axis represents mAU absorbance at 280 nm. Note: Both the retention volume and peak area of the digested sample is increased relative to the untreated sample, which suggests formation of several Mft1p-Thp2p complexes of lower overall molecular weight. Below the chromatogram is shown SDS-PAGE analysis (Coomassie-stained) of fractions B6-B1. Arrowheads are as in A and lane M shows marker proteins of known molecular weights. The gel shows protease resistant forms of both proteins. **C.** Purified samples of binary Mft1p$\Delta C_{248\text{-}392}$-Thp2p (lanes 1-2) and

Mft1p$\Delta C_{232-392}$-Thp2p (lanes 3-4) as identified by limited proteolysis. 10 µg (1x) or 20 µg (2x) was loaded in each lane. **D.** Mft1p$\Delta C_{336-392}$ (lanes 1–2) and Thp2p (lanes 3–4) purified in isolation and analysed by Coomassie-stained SDS-PAGE. In both C and D the lane marked M contains marker proteins of known molecular weights as indicated and arrowheads specify the position of indicated proteins. 10 µg (1x) or 20 µg (2x) was loaded in each lane. Protein complexes were purified in four steps: (i) Ni-NTA chromatography, (ii) TEV cleavage and Ni-NTA chromatography, (iii) Q-anion-exchange chromatography and (iv) gel-filtration chromatography, while the isolated proteins (Mft1p$\Delta C_{336-392}$ and Thp2p, respectively) were purified in three steps: (i) Ni-NTA chromatography, (ii) Q-anion-exchange chromatography and (iii) gel-filtration chromatography. Protein expression was performed using the *E. coli* Rosetta (DE3) strain.
(PDF)

Figure S4 SAXS curves and Guinier plots. SAXS data obtained for each sample as indicated. The Guinier plots (ln(s) versus s^2) are inset to show linearity. The corresponding R_g values are shown in Table S1.
(PDF)

Figure S5 Dimensionless Kratky plots. Kratky plots (s^2*I(s) versus s) are shown for each sample as indicated.
(PDF)

Figure S6 Analysis of Mft1p$\Delta C_{336-392}$ and Thp2p homodimers. For each sample (Mft1p$\Delta C_{336-392}$, green; Thp2p, orange/red), the asymmetric (non-constrained) and P2-constrained SAXS envelopes calculated using DAMMIF as well as GASBOR are shown in three perpendicular directions, along with the half-volume representing the monomeric protein (semi-transparent surface).
(PDF)

Figure S7 Multiphase-modeling in MONSA. **A.** Overlay of Mft1p$\Delta C_{336-392}$-Thp2p heterodimer representative models from DAMMIF (blue) and MONSA (grey) in three perpendicular views. **B.** Overlay of Mft1p$\Delta C_{336-392}$-Thp2p-Tho2p$\Delta C_{1274-1597}$ heterotrimer representative models from DAMMIF (magenta) and MONSA (grey) in three perpendicular views.
(PDF)

Figure S8 Dummy-atom models based on SAXS data from the trimeric THO complex. **A.** Filtered average model based on 11 dummy-atom models fitted to the data with a mean Normalised Spatial Discrepancy (NSD) value of 1.108 and variation of 0.056. **B.** Reference dummy-atom model showing the highest resemblance to the remaining models with an average NSD of 1.028. **C.** Dummy-atom model showing the second lowest NSD (1.042). **D.** Dummy-atom model showing the third lowest NSD (1.056). **E.** Overlay of the models in A-D. Some variation is observed in the part of the trimer corresponding to Tho2p (circled).
(PDF)

Table S1 Molecular parameters for each sample analysed by SAXS. The table shows the SAXS-derived parameters, R_g, D_{max}, and MW. The theoretical MW is the molecular weight calculated based on amino acid sequence and the numbers in parentheses are the corresponding homodimer masses. Experimentally determined MWs were based on comparisons with a BSA standard sample (*based on Porod volume).
(PDF)

Acknowledgments

We are thankful to beamline personnel at the X33, P12 and I911-4 beam lines for help in connection with the SAXS data collection and Professor Torben Heick Jensen for providing yeast strains used for expression cloning.

Author Contributions

Conceived and designed the experiments: JBP LES ED TB DEB. Performed the experiments: JBP LES EDA ED TB. Analyzed the data: JBP LES TB DEB. Contributed reagents/materials/analysis tools: TB. Wrote the paper: JBP TB DEB.

References

1. Kohler A, Hurt E (2007) Exporting RNA from the nucleus to the cytoplasm. Nature reviews Molecular cell biology 8: 761–773.
2. Houseley J, LaCava J, Tollervey D (2006) RNA-quality control by the exosome. Nature reviews Molecular cell biology 7: 529–539.
3. Meinel DM, Burkert-Kautzsch C, Kieser A, O'Duibhir E, Siebert M, et al. (2013) Recruitment of TREX to the Transcription Machinery by Its Direct Binding to the Phospho-CTD of RNA Polymerase II. PLoS Genet 9: e1003914.
4. Strasser K, Masuda S, Mason P, Pfannstiel J, Oppizzi M, et al. (2002) TREX is a conserved complex coupling transcription with messenger RNA export. Nature 417: 304–308.
5. Hurt E, Luo MJ, Rother S, Reed R, Strasser K (2004) Cotranscriptional recruitment of the serine-arginine-rich (SR)-like proteins Gbp2 and Hrb1 to nascent mRNA via the TREX complex. Proceedings of the National Academy of Sciences of the United States of America 101: 1858–1862.
6. Zenklusen D, Vinciguerra P, Wyss JC, Stutz F (2002) Stable mRNP formation and export require cotranscriptional recruitment of the mRNA export factors Yra1p and Sub2p by Hpr1p. Molecular and cellular biology 22: 8241–8253.
7. Chanarat S, Seizl M, Strasser K (2011) The Prp19 complex is a novel transcription elongation factor required for TREX occupancy at transcribed genes. Genes & development 25: 1147–1158.
8. Aguilera A (2005) Cotranscriptional mRNP assembly: from the DNA to the nuclear pore. Current opinion in cell biology 17: 242–250.
9. Huertas P, Aguilera A (2003) Cotranscriptionally formed DNA:RNA hybrids mediate transcription elongation impairment and transcription-associated recombination. Molecular cell 12: 711–721.
10. Rougemaille M, Dieppois G, Kisseleva-Romanova E, Gudipati RK, Lemoine S, et al. (2008) THO/Sub2p functions to coordinate 3'-end processing with gene-nuclear pore association. Cell 135: 308–321.
11. Chavez S, Garcia-Rubio M, Prado F, Aguilera A (2001) Hpr1 is preferentially required for transcription of either long or G+C-rich DNA sequences in Saccharomyces cerevisiae. Molecular and cellular biology 21: 7054–7064.
12. Chavez S, Beilharz T, Rondon AG, Erdjument-Bromage H, Tempst P, et al. (2000) A protein complex containing Tho2, Hpr1, Mft1 and a novel protein, Thp2, connects transcription elongation with mitotic recombination in Saccharomyces cerevisiae. The EMBO journal 19: 5824–5834.
13. Pena A, Gewartowski K, Mroczek S, Cuellar J, Szykowska A, et al. (2012) Architecture and nucleic acids recognition mechanism of the THO complex, an mRNP assembly factor. The EMBO journal 31: 1605–1616.
14. Flemming D, Thierbach K, Stelter P, Bottcher B, Hurt E (2010) Precise mapping of subunits in multiprotein complexes by a versatile electron microscopy label. Nature structural & molecular biology 17: 775–778.
15. Konarev PV, Petoukhov MV, Volkov VV, Svergun DI (2006) ATSAS 2.1, a program package for small-angle scattering data analysis. J Appl Cryst 39: 277–286.
16. Konarev PV, Volkov VV, Sokolova AV, Koch MHJ, Svergun DI (2003) PRIMUS: a Windows PC-based system for small-angle scattering data analysis. J Appl Cryst 36: 1277–1282.
17. Svergun DI (1992) Determination of the Regularization Parameter in Indirect-Transform Methods Using Perceptual Criteria. J Appl Cryst 25: 495–503.
18. Petoukhov MV, Konarev PV, Kikhney AG, Svergun DI (2007) ATSAS 2.1 - towards automated and web-supported small-angle scattering data analysis. J Appl Cryst 40: S223–228.
19. Franke D, Svergun DI (2009) DAMMIF, a program for rapid ab-initio shape determination in small-angle scattering. J Appl Cryst 42: 342–346.
20. Volkov VV, Svergun DI (2003) Uniqueness of ab initio shape determination in small-angle scattering. J Appl Cryst 36: 860–864.
21. Wriggers W (2010) Using Situs for the integration of multi-resolution structures. Biophysical Rev 2: 21–27.
22. Pettersen EF, Goddard TD, Huang CC, Couch GS, Greenblatt DM, et al. (2004) UCSF chimera - A visualization system for exploratory research and analysis. J Comp Chem 25: 1605–1612.
23. Svergun DI, Petoukhov MV, Koch MHJ (2001) Determination of domain structure of proteins from X-ray solution scattering. Biophys J 80: 2946–2953.

24. Svergun DI (1999) Restoring low resolution structure of biological macromolecules from solution scattering using simulated annealing. Biophys J 76: 2879–2886.
25. Linding R, Russell RB, Neduva V, Gibson TJ (2003) GlobPlot: Exploring protein sequences for globularity and disorder. Nucleic acids research 31: 3701–3708.
26. Homma K, Fukuchi S, Nishikawa K, Sakamoto S, Sugawara H (2012) Intrinsically disordered regions have specific functions in mitochondrial and nuclear proteins. Molecular bioSystems 8: 247–255.
27. Singh GP, Ganapathi M, Dash D (2007) Role of intrinsic disorder in transient interactions of hub proteins. Proteins 66: 761–765.
28. Perkins JR, Diboun I, Dessailly BH, Lees JG, Orengo C (2010) Transient protein-protein interactions: structural, functional, and network properties. Structure 18: 1233–1243.
29. Gwizdek C, Iglesias N, Rodriguez MS, Ossareh-Nazari B, Hobeika M, et al. (2006) Ubiquitin-associated domain of Mex67 synchronizes recruitment of the mRNA export machinery with transcription. Proceedings of the National Academy of Sciences of the United States of America 103: 16376–16381.
30. Ho Y, Gruhler A, Heilbut A, Bader GD, Moore L, et al. (2002) Systematic identification of protein complexes in Saccharomyces cerevisiae by mass spectrometry. Nature 415: 180–183.
31. Schneiter R, Guerra CE, Lampl M, Gogg G, Kohlwein SD, et al. (1999) The Saccharomyces cerevisiae hyperrecombination mutant hpr1Delta is synthetically lethal with two conditional alleles of the acetyl coenzyme A carboxylase gene and causes a defect in nuclear export of polyadenylated RNA. Molecular and cellular biology 19: 3415–3422.
32. Aguilera A, Klein HL (1990) HPR1, a novel yeast gene that prevents intrachromosomal excision recombination, shows carboxy-terminal homology to the Saccharomyces cerevisiae TOP1 gene. Molecular and cellular biology 10: 1439–1451.
